# Centimeter‐Scale Self‐Assembling Tendon Organoids Drive Tissue Regeneration

**DOI:** 10.1002/advs.202509453

**Published:** 2025-08-29

**Authors:** Tianshun Fang, Hong Zhang, Yuanhao Xie, Xiongfeng Li, Xi Liu, Zichen Wang, Yiwen Xue, Xiaohui Xia, Zetao Wang, Tingyun Lei, Ruifu Lin, Weiliang Shen, Bingbing Wu, Yishan Chen, Yanan Du, Xiao Chen, Zi Yin

**Affiliations:** ^1^ Department of Orthopedic Surgery of Sir Run Run Shaw Hospital, and Liangzhu Laboratory Zhejiang University School of Medicine Hangzhou 310058 China; ^2^ Dr. Li Dak Sum & Yip Yio Chin Center for Stem Cells and Regenerative Medicine and Institute of Cell Biology, Zhejiang University School of Medicine Hangzhou 310058 P. R. China; ^3^ Center for Rehabilitation Medicine Rehabilitation & Sports Medicine Research Institute of Zhejiang Province Department of Rehabilitation Medicine Zhejiang Provincial People's Hospital Affiliated People's Hospital Hangzhou Medical College Hangzhou Zhejiang 310014 China; ^4^ Key Laboratory of Motor System Disease Research and Precision Therapy of Zhejiang Province Hangzhou Zhejiang Province 310058 China; ^5^ Department of Sports Medicine & Orthopedic Surgery the Second Affiliated Hospital, and Liangzhu Laboratory Zhejiang University School of Medicine Hangzhou 310058 China; ^6^ Huzhou Central Hospital Zhejiang University School of Medicine Huzhou Zhejiang 313000 China; ^7^ International Institutes of Medicine The 4th Affiliated Hospital of Zhejiang University School of Medicine Hangzhou Zhejiang 322000 China; ^8^ Zhejiang University‐University of Edinburgh Institute Zhejiang University International Campus Haining 314400 China; ^9^ School of Biomedical Engineering Tsinghua‐Peking Joint Center for LifeSciences Tsinghua University Beijing 100084 China

**Keywords:** organoid, stem cell, tendon, tissue engineering

## Abstract

As a cell‐deficient tissue, the scarcity of endogenous stem cells significantly hampers the regeneration of tissue structure and restoration of motor function following tendon injury. To engineer large‐scale transplantable stem cell‐derived organoids in vitro would show tremendous potential in regenerative medicine. Here, by optimizing chemical signals and mimicking tendon extracellular matrix, transplantable tendon organoids exceeding 3 cm in human tissue‐scale dimensions are ultimately developed. This strategy empowers tendon organoids, with high cellular viability, proliferation, tenogenic phenotype, and remarkable enhancements in extracellular matrix (ECM) production enabled self‐assembly. At the single‐cell level, the majority cells in tendon organoids successfully achieve precise tendon‐specific lineage differentiation in vitro while retaining the exceptional regenerative capacity characteristic of fetal tendons. In a tendon defect model, the organoids increase the retention rate of stem cells by 7.9 times at 4 weeks and initiate the formation of a denser repaired tendon with enhanced mechanical properties. Overall, an efficient construction of centimeter‐scale human tendon organoids with superior regenerative potential is achieved, providing a promising strategy for the regenerative medicine.

## Graphical abstract

Centimeter‐scale tendon organoids are engineered by mimicking the extracellular matrix and optimizing chemical cues to guide tendon stem/progenitor cells. These large, self‐assembling constructs overcome conventional size limitations and exhibit enhanced tenogenic differentiation, extracellular matrix production, and mechanical resilience. Their tissue‐scale dimensions enable structural integration and functional tendon repair in vivo, offering a clinically relevant strategy for transplantable organoid‐based regeneration.

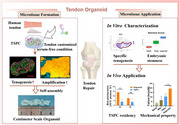

## 1 Introduction

Stem cell based engineered organ models represent a promising approach in regenerative medicine.^[^ ^1^ ^]^ Organoids, derived from patient‐specific stem cells, have demonstrated remarkable transplantation and repair capabilities in various studies involving the intestine,^[^ ^2^ ^]^ liver,^[^ ^3^ ^]^ skin,^[^ ^4^ ^]^ and pancreatic islets.^[^ ^5^ ^]^ Organoids have advantages like avoiding donor shortages in organ transplants, mimicking the structure and function of real organs, and minimizing immune rejection, which makes them highly applicable in regenerative medicine and holds great potential for future developments.^[^ ^6^ ^]^ However, despite their promise, organoids have not yet been adopted as substitutes for organ transplantation in clinical practice. A significant challenge lies in the substantial size discrepancy between stem cell‐derived organoids and human tissues or organs.^[^ ^7^, ^8^ ^]^ For instance, the large reported cartilage microtissues measure approximately 3.25 ± 0.05 millimeters, while defect areas in articular cartilage are often in the centimeter range.^[^ ^9^ ^]^ Similarly, existing liver organoids are only in the millimeter range, far from sufficient for liver transplantation.^[^ ^10^ ^]^ Therefore, developing functional organoids of human tissue size is crucial to advancing their application in organ transplantation therapies.^[^ ^11^ ^]^

The tendon, with its relatively simple cellular composition, serves as an ideal model for constructing large‐scale stem cell‐derived organoids. Its primary characteristic is an extracellular matrix (ECM)‐dominated composition, accounting for 60–85% of dry weight, with the ECM predominantly produced by the main parenchymal cell (tenocyte) within tendon.^[^ ^12^, ^13^, ^14^ ^]^ Tendon stem/progenitor cells (TSPCs) are a distinct population of stem cells found in tendons, characterized by their multipotency, self‐renewal capacity, and tenogenic phenotype, making them ideal seed cells for tendon formation and regeneration in vivo.^[^ ^15^ ^]^ Achieving exponential expansion of minimally sourced TSPCs obtained from biopsies or samples, is pivotal for the clinical application of stem cell‐based therapies.^[^ ^16^ ^]^ It has been reported that traditional serum‐containing medium may lead to a decline in cellular functionality.^[^ ^17^, ^18^ ^]^ Due to the batch effects and ingredient complexity of serum, which cannot be fully characterized or controlled, there is a clear need to develop a fully defined, customized culture medium. In vivo, the stable maintenance of cellular function depends on the precise spatiotemporal regulation of various physiological chemical signals. Harnessing bioactive molecules to replicate these in vivo signals for sustaining or reprogramming cell states holds considerable promise.^[^ ^19^ ^]^ For instance, Deng et al. employed five compounds to sustain the state of primary human hepatocytes in vitro over an extended period.^[^ ^20^ ^]^ Building on this concept, our developed high‐throughput screening platform identified small molecules and growth factors that promote both the proliferation and tenogenic differentiation of human TSPCs,^[^ ^21^, ^22^, ^23^, ^24^ ^]^ enabling the generation of a sufficient quantity of high‐quality cells. By precisely modulating key signaling pathways, it enhances tissue generation and is optimized for 3D culture systems, making it a powerful tool for tissue engineering and regenerative medicine.

However, due to the absence of a physical microenvironment, the exclusive use of chemical signals is insufficient to support the development of centimeter‐scale tendon‐like organoids. 3D cultures serve as an analog to recapitulate in vivo physiology and mimic natural tissue structures. Providing a supportive framework that simulates the interactions between stem cells and the ECM in vivo, while ensuring nutrient supply to the core regions, is critical for enabling the gradual expansion of microtissues to centimeter‐scale dimensions.^[^ ^25^, ^26^, ^27^ ^]^ Previously, TSPC cell sheets cultured in 2D monolayers encountered significant limitations, including inadequate nutrient penetration into inner regions.^[^ ^28^ ^]^ To overcome these challenges, we utilized a 3D porous microsphere (MSs) with high porosity and excellent biocompatibility due to their gelatin composition. The peptide sequences of arginine‐glycine‐aspartate within the MSs promote collagen assembly. These porous MSs enhance stem cell adhesion and provide robust mechanical support, optimal stiffness, and an enlarged specific surface area.^[^ ^22^, ^29^ ^]^ These attributes facilitate effective cell adhesion and ECM interactions, triggering positive differentiation signals in TSPCs. Moreover, their multi‐chamber structure permits nutrient diffusion into inner regions, supporting the formation of centimeter‐scale microtissues.

In this study, we develop a tendon organoid to provide high‐performance graft for tendon repair. By recapitulating the factors involved in the developmental process and employing a biomimetic physiological 3D microcarrier, we have developed an efficient strategy for constructing tendon organoids, enabling the production of centimeter‐scale macroscopic tissue resembling native tendons. This approach also offers new perspectives for engineering transplantable, large‐scale organoid constructs for clinical applications.

## 2 Results

### 2.1 Design and Fabrication of Tendon Organoids under Customized Culture Conditions

**Figure 1:**
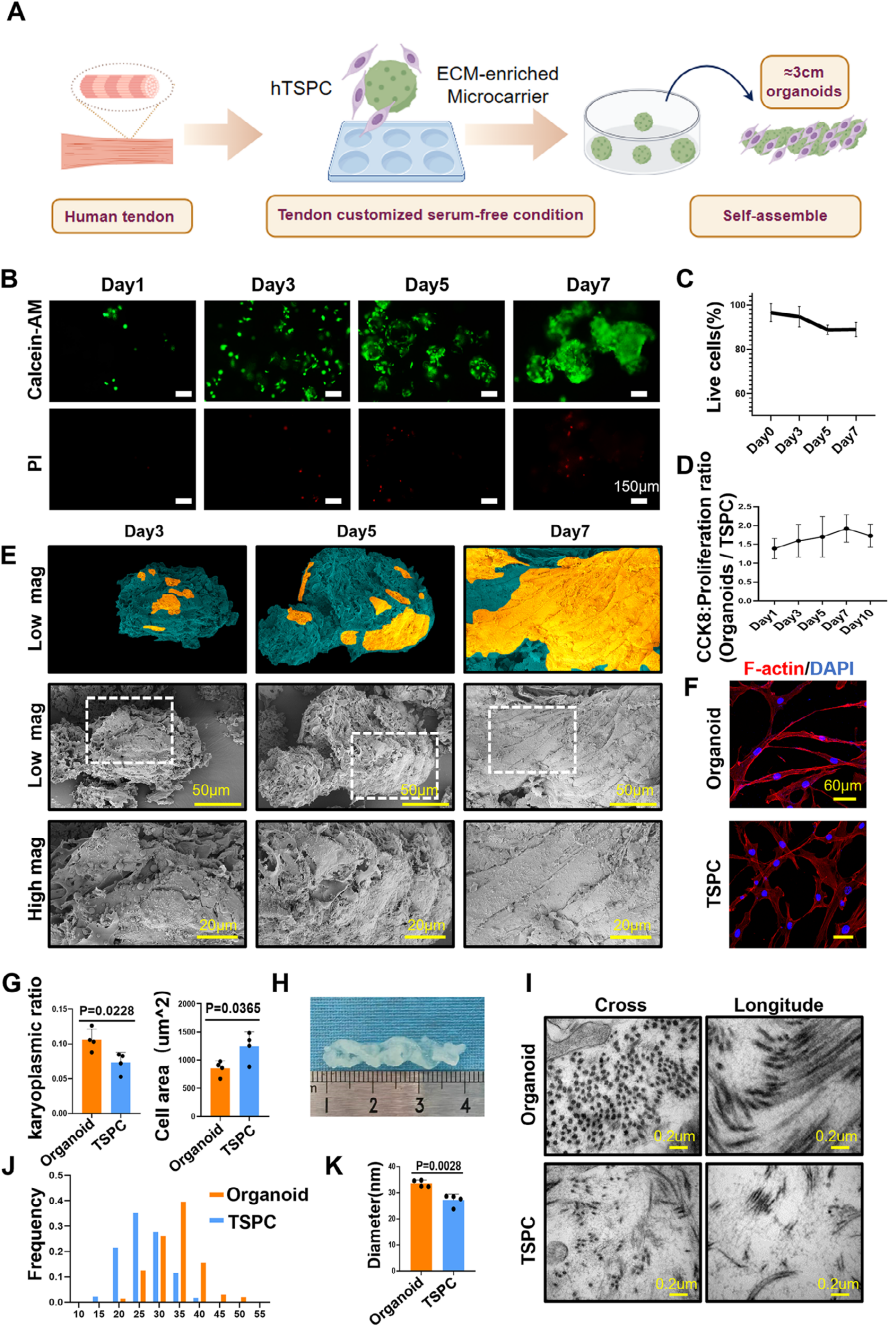
Characterization of tendon organoid with biomimetic microcarriers. A) Illustration of TSPCs based 3D organoid engineering. B,C) Live/dead cell staining was conducted to demonstrated the good cell viability of TSPCs in the organoid group at 1, 3, 5, and 7 d (*n* = 3). D) Cell proliferation rate of TSPCs in the organoid and control groups after cultured at 1, 3, 5 and 7 d. E) Scanning electron microscopy (SEM) images exhibit the self‐assembly and proliferation morphology of TSPCs in the organoid group at 3, 5, and 7 d. Green denotes the microcarrier scaffold, yellow indicates tendon cells. Scale bars: 20 µm, 50 µm. F,G) Representative confocal images of TSPCs in organoid (*n* = 4) and control conditions (*n* = 4) after 4 d which stained with F‐actin (phalloidin). Scale bars, 60 µm, the quantification of karyoplasmic ratio, cell area. H) General view of 3 cm tendon organoid cultured for 28 d. I–K) Transmission electron microscopy (TEM) exhibits longitudinal sections and cross‐sectional images of the collagen fibrils within organoid (*n* = 4) and control groups (*n* = 4) in vitro. Organoid groups have thicker diameters range of the collagen fibrils.

We have generated a centimeter‐scaled macroscopic tendon like tissue by developing an efficient tendon organoid construction strategy which leverages 3D MSs and biochemical culture conditions (**Figure** **1**A). The biomimetic ECM microcarriers serves as an exceptional scaffold for promoting the aggregation of stem cells in vitro. The 3D MSs, comprised of gelatin, are equipped with interconnected micropore arrangements, housing pore sizes between 10–30 µm and nearing 200 µm in diameter, as revealed by SEM (S1A). This porous structure provides extensive space for cell adhesion and proliferation, enabling cells to secure sufficient growth nutrients while nurturing cell–cell and cell–ECM interactions, thus showcasing robust adhesion and super biocompatibility to TSPCs. Live/dead staining demonstrated good cell viability and propagation in the MSs (Figure 1B,C and Figure S1D, Supporting Information).

We have developed a tendon‐specific condition for organoid culture. From day 3 to day 14, TSPCs proliferation in the organoid group (in tendon‐specific conditions) consistently surpassed that of the traditional 2D culture group, with the number of cells expanding to 8.29 times, while the 2D TSPC group only showed an expansion by 3.29 times (Figure S1E, Supporting Information). Importantly, CCK8 assays performed without MSs digestion further confirmed that the proliferation activity of the organoid group was markedly higher than that of the TSPC group (Figure 1D). The nuclear staining with DAPI also visually demonstrates a substantial temporal increase in cell population (Figure S1B, Supporting Information). With an extended culturing period, TSPCs on MSs gradually merged and self‐organized to form tendon‐like organoids on a microscopic scale (Figure 1E and Figure S1C, Supporting Information). We also assessed the in vitro tendon‐forming ability of TSPCs organoids. TEM revealed a significant growth in collagen fiber assembly ability of the organoid, with fewer and slender collagen fibril observed in the 2D‐cultured TSPCs group (Figure 1I–K). F‐actin immunofluorescence staining unveiled that TSPCs in the organoid group had a less diffuse morphology, smaller cellular area and volume, and improved nucleo‐cytoplasmic ratio and sphericity relative to the TSPCs group (Figure 1F,G). After 28 d of culture under our customized conditions, a tendon‐like microtissue exceeding 3 cm in length was successfully engineered (Figure 1H).

The aforementioned results suggest that the tendon organoid is capable of achieving high cell viability, efficient cell expansion, and self‐assembly into microtissues in vitro. This highlights its potential for simulating tendon formation.

### 2.2 The Evaluation of Centimeter Scale Microtissue Aggregated by TSPC

**Figure 2:**
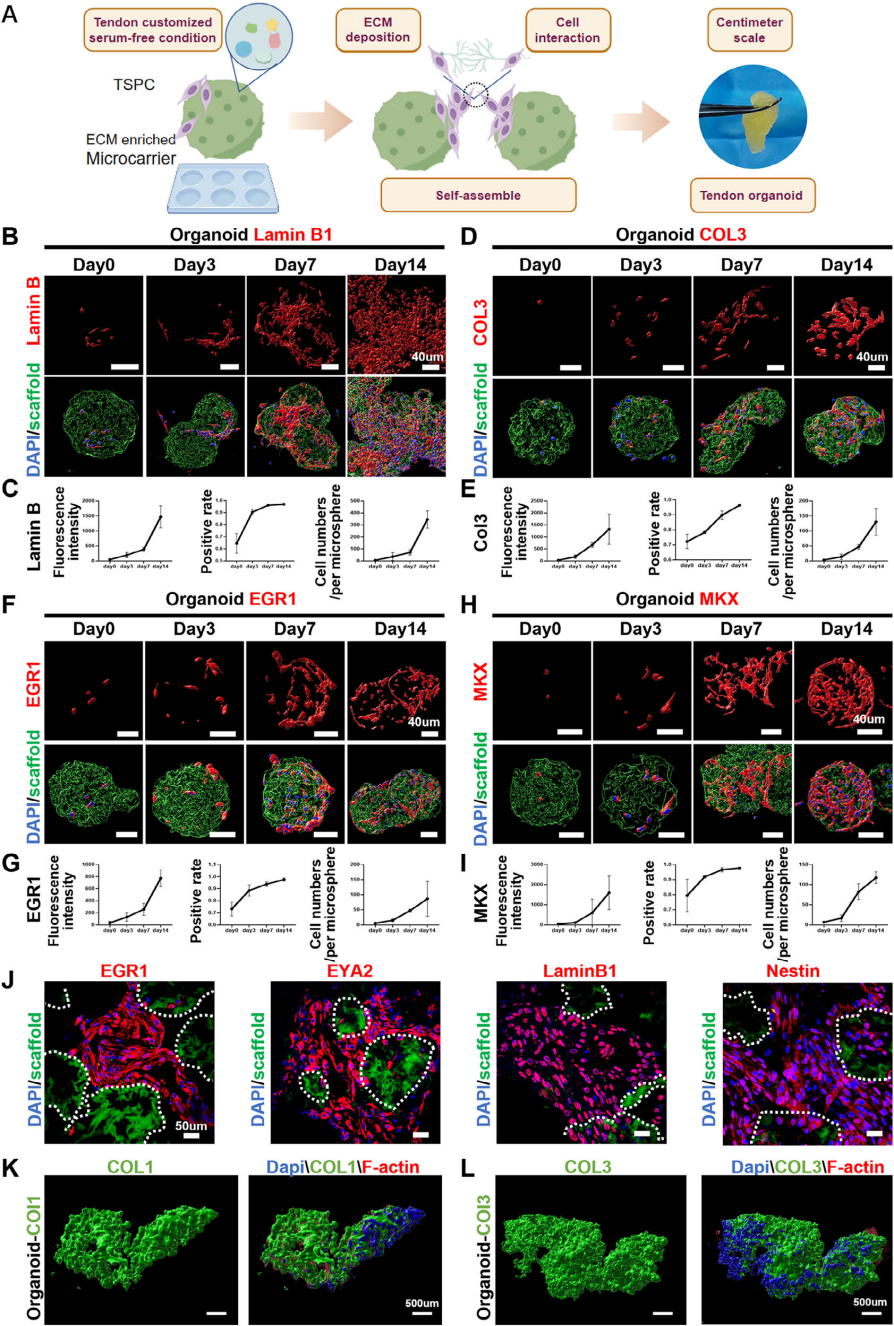
Tendon organoids exhibit spontaneous aggregation in vitro and manifest youthful characteristic. A) Schematic diagram and appearance of tendon organoids. B,C) Fluorescence microscopy images of TSPCs staining Lamin B1 cultured in organoid (*n* = 3) at day 0, 3, 7, and 14, and the quantification of mean fluorescence intensity, positive rate, and cell numbers/per microsphere, Scale bars: 40 µm. D,E) Fluorescence microscopy images of TSPCs staining Col3 cultured in organoid at day 0, 3, 7 and 14, and the quantification of mean fluorescence intensity, positive rate, and Cell numbers/per microsphere, Scale bars: 40 µm. F,G) Fluorescence microscopy images of TSPCs staining EGR1 cultured in organoid (*n* = 3) at day 0, 3, 7 and 14, and the quantification of mean fluorescence intensity, positive rate, and Cell numbers/per microsphere, Scale bars: 40 µm. H,I) Fluorescence microscopy images of TSPCs staining MKX cultured in organoid at day 0, 3, 7 and 14, and the quantification of Mean fluorescence intensity, positive rate, and Cell numbers/per microsphere, Scale bars: 40 µm. J) Representative confocal images of EGR1, EYA2, Lamin B and Nestin immunofluorescent staining of tendon organoid‐ without induction at day 14 in vitro. K,L) Light‐sheet microscopy images of the engineered organoid, with COL1 and COL3 staining, scale bar: 500 µm.

After 4 days in the organoid culture system, the cultured TSPCs initiated coagulation into mutually clustered structures, and these organoid structures significantly expanded over time, with their main axes exceeding 2 cm by day 21 (**Figure** **2A** and S2A, Supporting Information). The expression of Lamin B consistently increased significantly, with over 95% positive rate throughout the tendon formation process from day 1 to day 14, indicating that TSPCs could retain their youthful properties and stemness during the organoid formation (Figure 2B,C,J). In addition, tendon related markers such as COL3, EGR1, and MKX displayed pronounced expression within the organoids (Figure 2D–I,J and Figure S2D,E, Supporting Information). It is worth noting that under fluorescence microscopy, TSPCs can be found to proliferate rapidly and achieve fusion between adjacent microspheres. Light sheet microscopic also revealed high expression of COL1 and COL3 throughout the tendon organoid (Figure 2K, L). To assess the quality of the macroscopic tissue, histological analyses (hematoxylin and eosin (H&E) and Masson's trichrome staining) and immunostaining of relevant tenogenic and regenerative biomarkers were conducted on the self‐assembling tenogenic organoid at day 28. At the microscopic level, dense, longitudinally thread‐like collagen fibers were formed around the MSs, resembling the native collagen structure found in tendons in vivo (Figure S2B, Supporting Information). Interestingly, TSPCs were primarily situated on the MS surfaces and aggregate with one another. This intercellular fusion is speculated to be the primary driver promoting microtissue assembly (Figure S2C, Supporting Information).

These results revealed that the tendon organoid possesses the capability to form complete micro tendon tissue, while also manifesting typical tenogenic and youthful markers.

### 2.3 Tendon Organoids Exhibit Multipotency and Antisenescence Characteristics

The stem cell properties of TSPCs during tendon organoid expansion were assessed by evaluating multi‐lineage differentiation capacity and TSPCs surface marker expression. The TSPCs within the 3D organoids maintained their characteristic surface marker profile of CD105^+^, CD90^+^, and CD44^+^ even after extensive expansion, consistent with the previously reported stem cell surface marker expression (Figure S3A, Supporting Information). Our results revealed that the TSPCs in organoids showcased significantly enhanced tenogenic, osteogenic, and chondrogenic capabilities. However, no notable differences were found when considering adipogenic capacity augmentation (Figure S3B, Supporting Information). Interestingly, when passaged in vitro to the 8th generation, the organoids displayed substantially decreased expression of β‐galactosidase (β‐gal) and remarkably increased expression of youth‐associated markers (Ki67, Lamin B) and TSPCs related marker (Nestin), indicating that the organoids possess anti‐aging and rejuvenating characteristics (Figures S3C—G and S4A,C,D, Supporting Information). Further, using 5‐ethyl‐2′‐deoxyuridine (EdU) to label proliferating cells indicated that organoids contain a higher number of proliferating TSPCs (Figures S3E and S4B, Supporting Information). Compared with the 2D TSPCs cultured group, qPCR further confirmed organoids had higher expression of tenogenic and regenerative markers including *SCX, THBS4, BGN, EYA2, TNMD*, and *Ki67* (Figure S3H, Supporting Information).

These results underscore that organoids not only retain stem cell potential following successive passages, but they also exhibit superior multi‐lineage differentiation potential alongside rejuvenating characteristics.

### 2.4 Transcriptional Profiling Reveals Strong Tenogenic Differentiation in TSPC‐Derived Organoid

**Figure 3:**
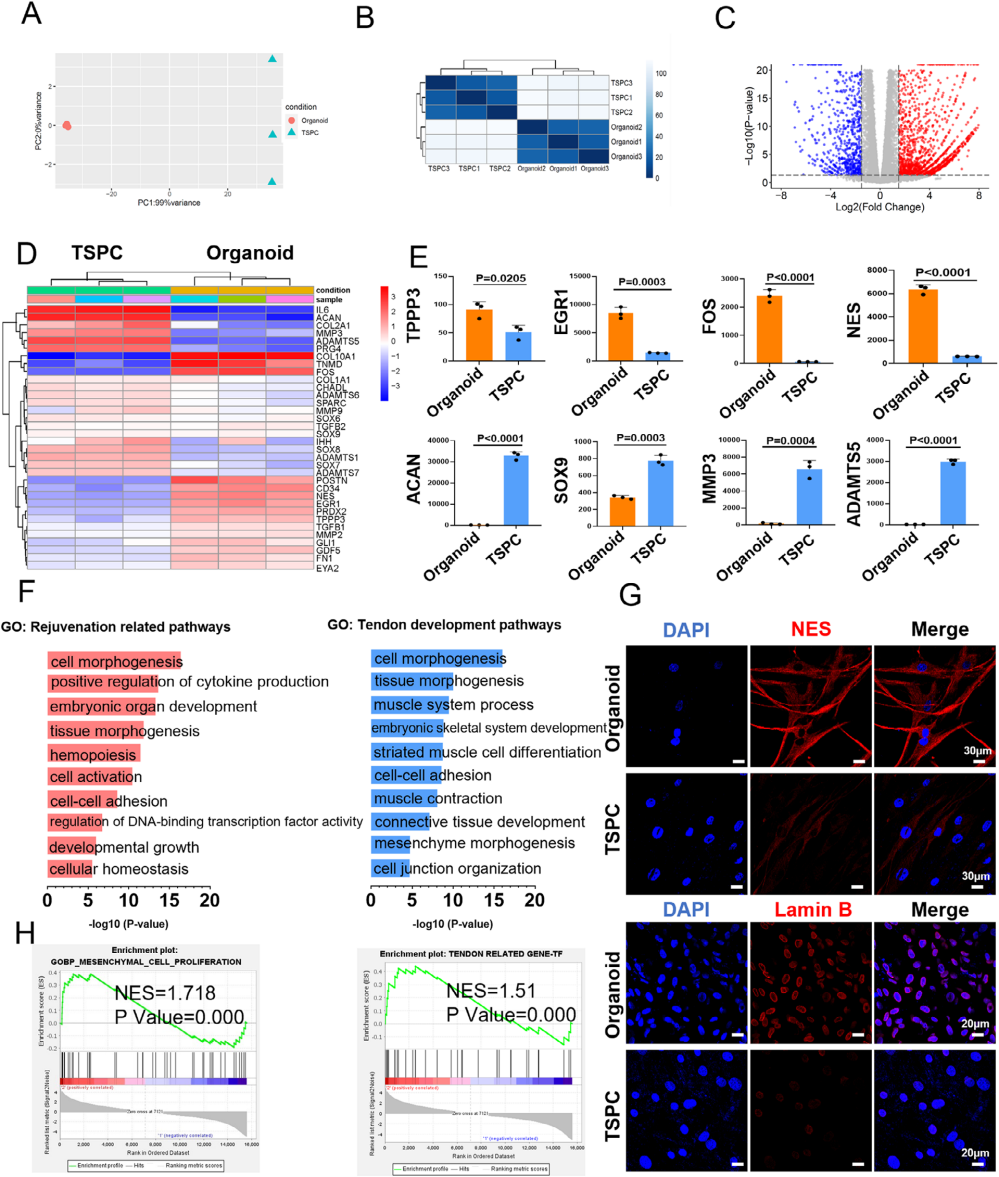
TSPCs organoids in sustaining differentiation and self‐renewal processes at the transcriptional level. A) A principal component analysis (PCA) plot was generated to visualize the clustering of organoid group (*n* = 3) and was distinctly separated from the control group (*n* = 3) at 3 d. B) Heatmap was constructed to display the sample‐to‐sample distances utilizing rlog‐transformed values. C) Volcano plot of the gene expression differences between organoid and control‐cultured TSPCs (organoid vs TSPC; log2 fold change >1.5, < −1.5; *p* < 0.0500; *n* = 3). D) Heatmap of the differentially expressed genes (DEGs) related to tenogenic, ossification and inflammation, with the color scale ranges from blue to red, representing low to high gene expression levels, respectively. E) mRNA expression of the *TPPP3, EGR1, FOS, NES, ACAN, SOX9, MMP3* and *ADAMTS5* of TSPCs after being cultured in the organoid and control (*n*  =  3). F) Analysis of enriched biological processes (GO) in organoid group compared with control. H) Gene set enrichment analysis (GSEA) of the tendon related genes. NES = normalized enrichment score; FDR = false discovery rate. G) Immunofluorescence staining for Nes and Lamin B in TSPCs isolated from the organoid and control groups on day 4. (TSPCs isolated from organoid VS Conventionally cultured TSPCs) Scale bars: 30 µm, 20 µm.

To examine the gene expression patterns in TSPC organoids, we utilized bulk RNA‐Seq to analyze the gene expression transition of tendon organoid and 2D cultured TSPCs. Principal component analysis (PCA) and heat maps revealed that the organoid group samples clustered together and distinct from the TSPC control group (**Figure** **3**A,B). We identified 1136 down‐regulated genes and 429 upregulated genes in the organoid group (log2‐fold change > 1.5 or < −1.5; *p* < 0.0500) (Figure 3C). To further explore the role of signal transduction in tenogenic differentiation and tendon microtissue composition within the organoid culture system, we performed gene ontology (GO) analysis of differentially expressed genes (DEGs) (Figure 3D). Key biomarkers associated with tenogenesis and proliferation (such as *TPPP3, EGR1, FOS*, and *N* *ES*) were upregulated in organoids, while cartilage markers (like *ACAN* and *SOX9*) linked to tendon heterotopic ossification, and degradation markers (such as *MMPs* and *ADAMTS*) showed significant decrease (Figure 3E). Western blot analysis confirmed marked upregulation of the tendon markers MKX, the rejuvenation marker LAMINB, and the TSPC marker NES in the organoid group (Figure S4E, Supporting Information). In addition, GO pathway analysis further disclosed significant gene enrichment in both rejuvenation‐related pathways (cell morphogenesis, embryonic organ development, and developmental growth) and tendon development pathways (tissue morphogenesis, and mesenchyme morphogenesis) (Figure 3F and Figure S5B, Supporting Information). Notably, due to the limited representation of tendon‐specific terms in current gene ontology databases, the enrichment of tendon‐related processes tends to be captured under broader or muscle‐associated terms that share overlapping regulatory genes. Gene set enrichment analysis (GSEA) further confirmed enrichment of the mesenchymal_cell_proliferation pathway (NES = 1.718, *P* < 0.001) and tendon‐related gene TF (NES = 1.51, *P* < 0.001) in organoids (Figure 3H). In addition, TSPCs tendon organoids also enriched embryonic skeletal joint development (NES = 1.766, *P* < 0.001), positive regulation of stress fiber assembly (NES = 1.903, *P* < 0.001) and proteoglycan biosynthetic process (NES = 1.881, *P* < 0.001) pathways (Figure S5A, Supporting Information). Subsequent evaluation of the tendon‐forming capacity of TSPCs isolated from the tendon organoids via immunofluorescence staining detected significant increases in tendon‐specific marker (Nestin) and rejuvenation marker (Lamin B1) (Figure 3G).

**Figure 4:**
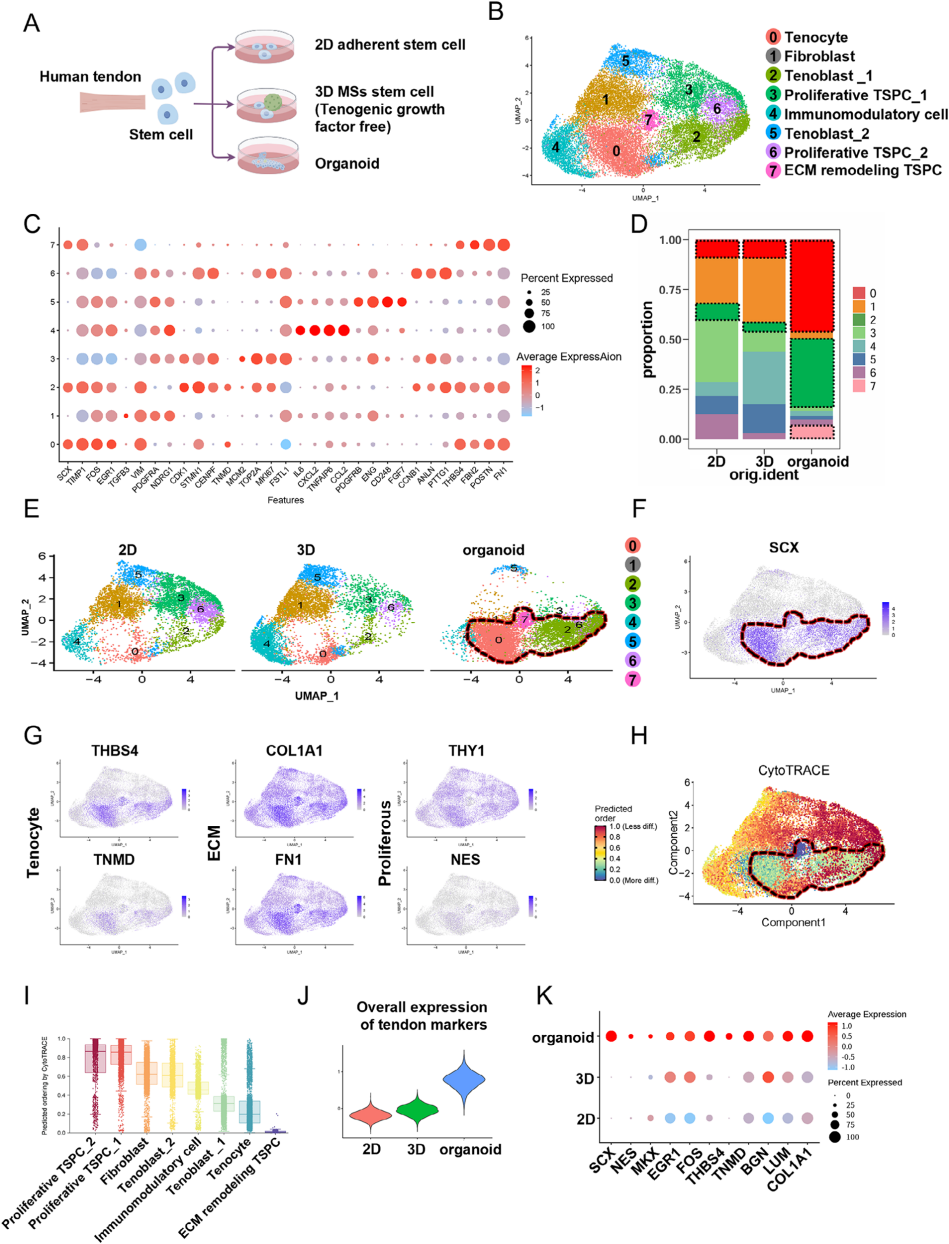
The organoids demonstrated excellent tendon maturity, achieving in vitro differentiation of the SCX lineage. A) Illustration of TSPCs cultured in 3 groups: organoid, 2D cultured TSPCs, and 3D MSs cultured TSPCs. B) UMAP visualization of the integrated data from the 2D, 3D, and organoid groups. C) Dot plot of marker genes for each cluster. D,E) Proportional distribution chart of cell subpopulations. F,G) Feature plot of marker genes. H,I) Cytotrace differentiation potential plot. J) Overall expression score of tendon cells. (K) Dot plot of overall tendon markers expression.

These findings underscore the profound potential of TSPCs organoids in sustaining differentiation and self‐renewal processes at the transcriptional level. Furthermore, they highlight the advantages that organoids offer in biomimicking the intricate formation and regenerative capabilities of complex tendon microtissues in a controlled in vitro environment.

### 2.5 The Organoids Demonstrated Excellent Tendon Maturity and Achieved In Vitro Differentiation of Tendon‐Specific Lineage

To characterize the cell state within organoids, we performed scRNA‐seq on organoids, as well as 2D cultured TSPCs, and 3D MSs cultured TSPCs (without serum‐free conditions) (Figure 4A). The cells were classified into eight distinct clusters: Tenocyte *(SCX, EGR1*), Fibroblast (*PDGFRA, TGFB3*), Tenoblast_1 (*CDK1, STMN1*), Proliferative TSPC (*MCM2, TOP2A*), Immunomodulatory cell (*IL6, CXCL2*), Tenoblast_2 (*FGF7, PDGFB*), and ECM remodeling TSPC (*FN1, FBN2*), based on the most significantly enriched genes within each group (Figure 4B,C). The organoid‐derived TSPCs displayed notable enrichment in clusters corresponding to Tenocytes (cluster 0) and Tenoblasts (cluster 2). In contrast, the 2D culture and 3D MS culture groups both showed an enrichment of immune marker‐expressing immunomodulatory cells (cluster 4) rather than tenocyte‐related subpopulations. This suggests that the organoid system more closely mimics the in vivo‐like differentiation process, where TSPCs gradually transition to mature tenocytes (Figure 4D,E). Additionally, the organoid group exhibited significantly higher expression of the tendon‐specific marker *SCX* compared to the 2D cultured and 3D MS cultured groups (Figure 4F). Further, the expression of other tendon markers (*TNMD, THBS4)* and ECM components (*COL1A1, FN1*) was markedly upregulated in the organoid dominant cell clusters, whereas the upregulation of stem cell markers (*THY1, NES*) was less pronounced (Figure 4G). The proportion of cell subpopulations with high expression of tendon‐associated genes (Tenocyte and Tenoblast_1) increased significantly, from 13.46% in the 2D group and 13.65% in the 3D MSs‐only group, to 80.67% in the organoid group. Cytotrace analysis, used to evaluate cellular differentiation potential, revealed that the organoid‐derived tenocyte clusters exhibited the lowest degree of differentiation potential, reflecting a terminally differentiated state and highest maturity, which is closely resembling the in vivo tendon differentiation process (Figure 4H,I). Evaluation of key tendon markers, including *SCX, MKX, FMOD*, and *DCN*, showed that the organoid group exhibited significant upregulation tendon markers. In contrast, 3D MS culture slightly increased the expression of *EGR1, FOS*, and *DCN*. The 3D MS culture did not show enrichment of critical tendon‐specific markers, such as *SCX, MKX*, and parenchymal tendon markers (*THBS4, COL1A1, BGN, TNMD*), as observed in the organoid group (Figure 4J,K).

These results highlight the ability of organoids to effectively induce tendon specific lineage differentiation in vitro, recapitulating the in vivo progression from stem cells to mature tenocytes, and further validate that our system as a robust model for tendon organoid studies.

### 2.6 Single‐Cell Transcriptomic Analysis Positions the Organoid Group as an Intermediate State Between In Vitro Cultured Cells and Native Tendon Tissue

**Figure 5:**
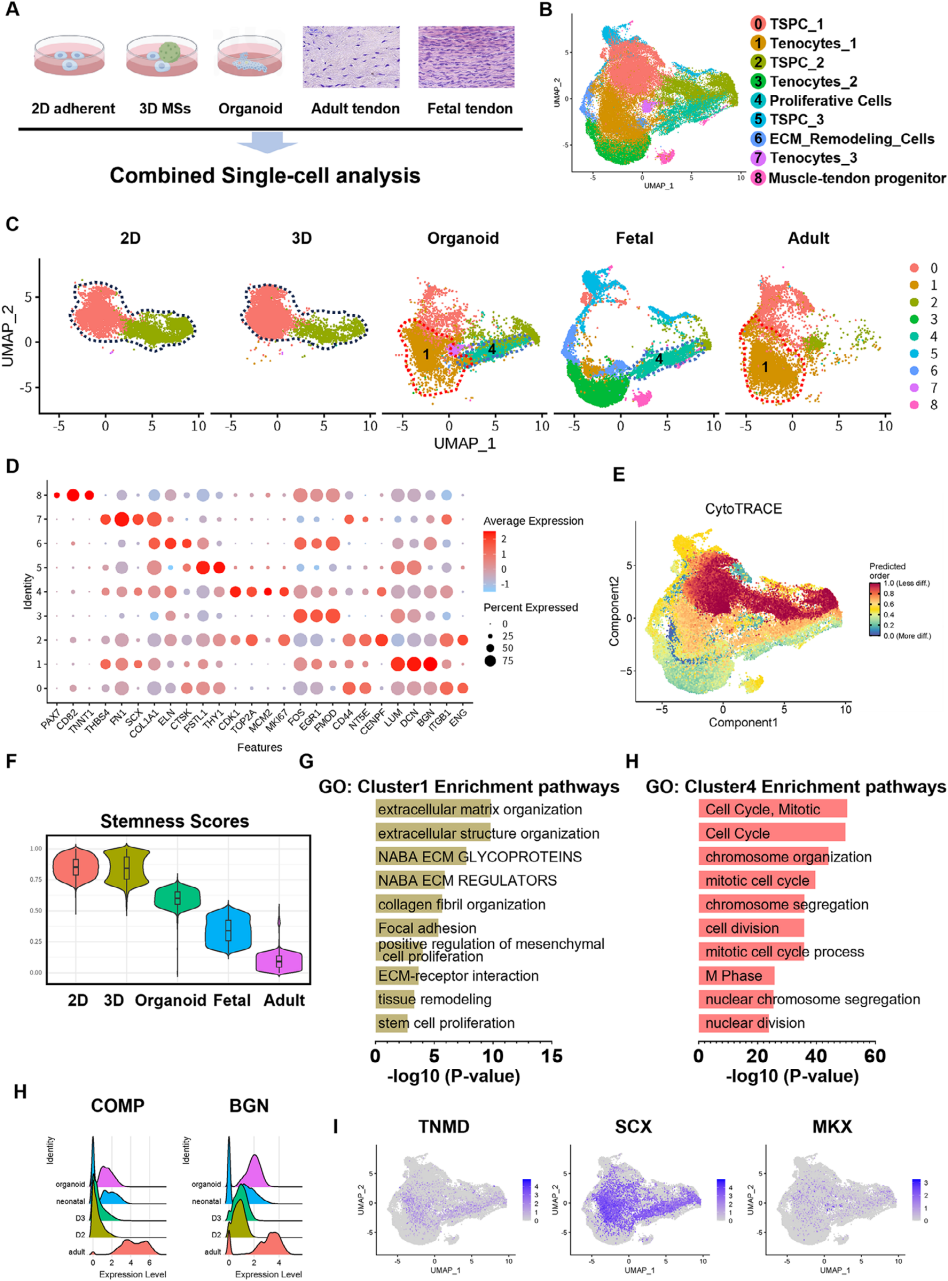
Single‐cell sequencing reveals that the organoid group represents a transitional state between in vitro cultured TSPCs and native human tendon tissue. A) Illustration of TSPCs cultured in 3 groups: organoid, human neonatal tendon, and human adult tendon. B) UMAP visualization of the integrated data from 2D, 3D scaffold‐only, the organoid group, tendon‐related subpopulations from tendon human neonatal and adult tendon. C) Proportional distribution chart of cell subpopulations of each sample. D) Dot plot of marker genes for each cluster. E) Cytotrace differentiation potential plot by clusters. F) Cytotrace differentiation potential plot by samples. G,H) GO enrichment of cluster 1 and cluster 4. H) RidgePlot of marker genes. I) Feature plot of marker genes.

To comprehensively evaluate the molecular similarity between tendon organoids and native tendon tissue, we performed an integrated single‐cell RNA sequencing (scRNA‐seq) analysis across five groups, including organoid‐derived TSPCs, 2D‐cultured TSPCs, 3D MSs‐only cultured TSPCs, fetal tendons, and adult tendons (**Figure** **5**A). Human tendon samples were first clustered based on key tendon markers (*SCX, EGR1, FOS, FMOD, BGN, DCN, LUM, COL1A1, THBS4, MKX*) to identify tendon‐associated subpopulations (Figure S5C, Supporting Information). Integration with organoid samples revealed 9 distinct clusters: TSPC_1 (*ENG, ITGB1*), Tenocytes_1 (*BGN, DCN, LUM*), TSPC_2 (*CENPF, NT5E, CD44*), Tenocytes_2 (*FMOD, EGR1, FOS*), Proliferating cells (*MKI67, MCM2, TOP2A, CDK1*), TSPC_3 (*THY1, FSTL1*), ECM remodeling cells (*CTSK, ELN, COL1A1*), Tenocyte_3 (*SCX, FN1, THBS4*), Muscle‐tendon progenitor (*TNN1, CD82, PAX7*). In the integrated single‐cell clustering, 2D and 3D scaffold‐only groups remained largely confined to two TSPC‐related clusters. In contrast, the organoid group occupied an intermediate transcriptional state between in vitro cultured cells and native tendon tissue. Notably, the organoids contained multiple mature tendon‐related subpopulations, including a mature tenocyte cluster (Tenocyte_1) closely resembling adult tendon tissue, as well as a proliferative subpopulation highly similar to that of fetal tendons (Figure 5B–D). Cytotrace‐based stemness scoring revealed that the tenocyte_1 subpopulation—shared between organoids and mature tendon tissue—exhibited low stemness scores, consistent with the differentiated state of native tenocytes (Figure 5E). Moreover, the overall stemness profile of the organoids was lower than that of 2D and 3D cultured TSPCs, slightly higher than fetal tendon, and notably higher than adult tendon. These findings further support that the organoids represent a transitional state during TSPC maturation toward functional tendon tissue (Figure 5F). GO analysis further revealed that the Tenocyte_1 cluster, shared between organoids and mature tendon tissue, was primarily enriched in pathways related to extracellular matrix organization and collagen fibril formation, characteristic of mature tendon function. In contrast, the proliferative cluster shared between organoids and fetal tendon tissue exhibited high enrichment in cell proliferation and cell cycle‐related pathways (Figure 5G,H). Ridge Plot analysis of COMP expression demonstrated levels in the organoid group comparable to those in the fetal tendon, lower than adult tendon, but substantially higher than in the 2D and 3D TSPC groups (Figure 5H). Key markers involved in tendon maturation—including *SCX*, *MKX*, and *TNMD*—were enriched across both the tenocyte_1 and proliferative cell clusters within the organoid group rather than the 2D and 3D TSPC groups (Figure 5I).

In summary, organoids represent a transitional state between in vitro cultured TSPCs and native tendon tissues, exhibiting both enhanced maturity and retained proliferative potential, thereby providing a valuable model for tendon regeneration and tissue engineering applications.

### 2.7 The Tendon Organoid Enhances Self‐Assembly by Enriching ECM Components

**Figure 6:**
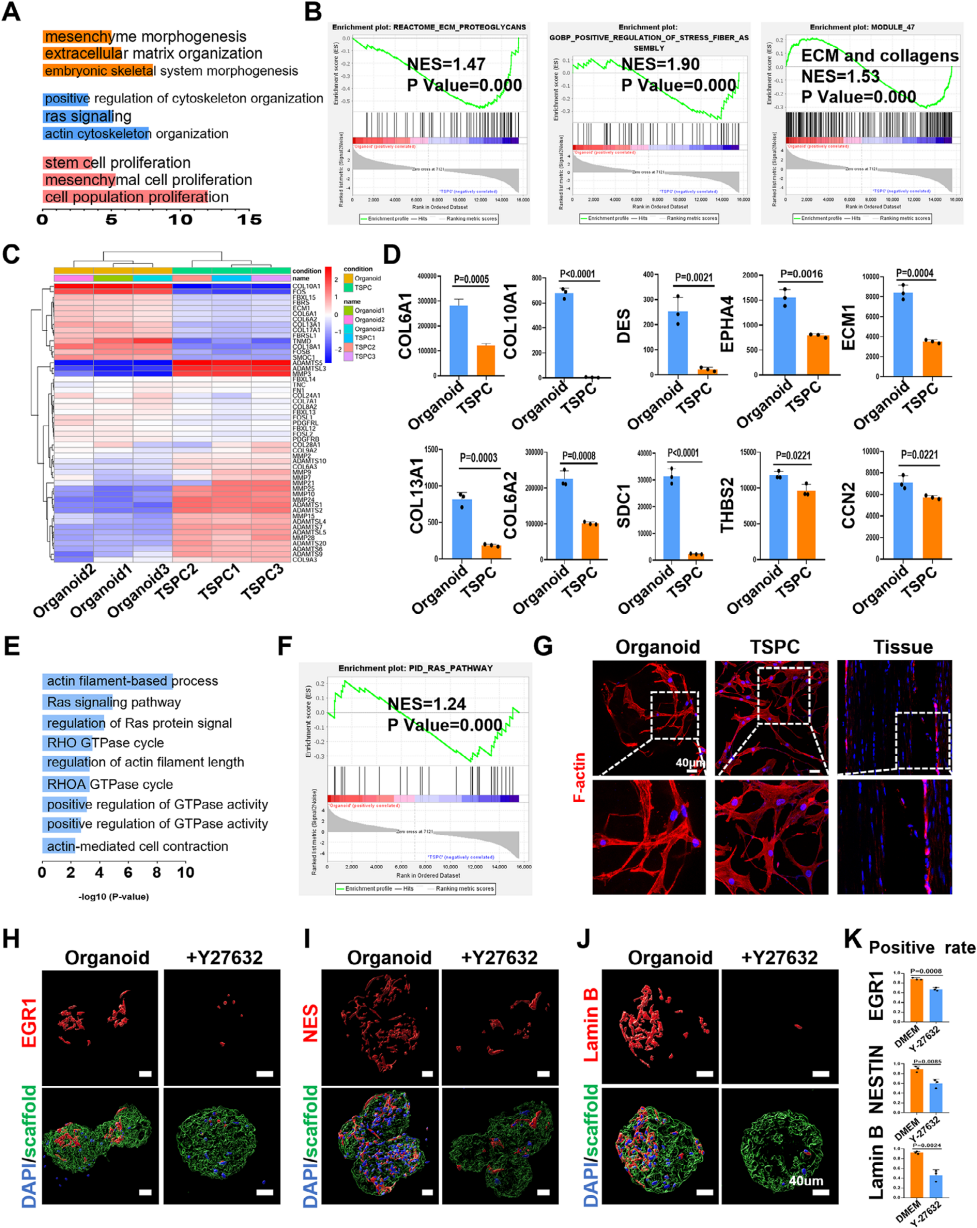
Organoids enrich ECM components and activate F‐actin to promote tissue formation. A) Analysis of enriched biological processes (GO) of ECM and stem cell in organoid group compared with control. B) GSEA of the ECM related pathways. C) Heatmap of the differentially expressed genes (DEGs) related to ECM, with the color scale ranges from blue to red, representing low to high gene expression levels, respectively (*n* = 3). E) mRNA expression of the *COL6A1, COL10A1, DES, EPHA4, ECM1, COL13A1, COL6A2, SDC1, THBS2* and *CCN2* of TSPCs after being cultured in the organoid and control (*n*  =  3). E) Analysis of enriched biological processes (GO) of actin in organoid group compared with control. B) GSEA of the cytoskeleton related pathways. G) Cytoskeletal morphology of TSPCs in organoids, TSPC group and in vivo. D–G) Representative confocal images of EGR1, Nes and Lamin B immunofluorescent staining between DMEM and Y27632 treatment, and the quantification of positive rate.

We then further analyzed RNA‐seq to gain deeper insights into the underlying mechanisms responsible for maintaining cell function and preventing phenotypic loss. The GO analysis revealed activation of stem cell‐related pathways and ECM related pathways (extracellular matrix organization) in tendon organoids (**Figure** **6**A). GESA signally demonstrated that the genes associated with ECM‐pathway and collagen related pathways were higher in the tendon organoid group (Figure 6B). In addition, key biomarkers associated with ECM sedimentation and collagen formation (such as *ECM1, COL6A1, COL10A1* and *FOS*) were upregulated in organoids, while matrix degradation markers (like *MMP9, ADAMTS1* and *ADAMTS2*) linked to tendon fibrosis showed significant decrease (Figure 6C,D). We conducted further analysis to elucidate the intricate pathways through which the ECM formation exerts control over fundamental processes, including cytoskeleton maintenance, metabolism, and organoid expansion. During the in vitro culture, we observed that the cell morphology of the tendon organoid was more closely resembled in natural in vivo state compared to control group (Figure 1J–L). The GO and GSEA analysis also indicated that the cytoskeleton pathway is significantly enriched in tendon organoids (Figure 6E,F). Compared with the 2D cultured TSPC group, the skeletal morphology of stem cells in the organoid group was more consistent with the elongated morphology of in vivo tissues (Figure 6G). Collectively, these findings suggest that cytoskeletal mechanical signals likely play a role in the formation of tendon organoids. To examine and validate this hypothesis, we treated tendon organoids with 5 uM Y‐27632 (ROCK inhibitor) for 24 h from day 4 and collected samples on day 5. As anticipated, ROCK were involved in mechanical signaling transduction to modulate cell morphology, we observed a distinct diffusion pattern in cell morphology, characterized by significantly larger cell area and volume and a reduction in the nucleo‐plasmic ratio and sphericity after ROCK inhibition (Figure S6A, Supporting Information). In the Y27632‐treated group, the expression levels of tendon‐related markers (EGR1, NES, and EYA2) decreased. Additionally, the expression of youthfulness marker Lamin B was found to be reduced following cytoskeletal inhibition (Figure 6H–K and Figure S6B, Supporting Information).

In summary, our study reveals that the activity and tendon lineage differentiation potential of organoid is contingent upon the ECM enrich signals, and this effect further mediates cell assembly and differentiation potential via the downstream maintenance cytoskeleton signaling pathway.

### 2.8 TSPCs Organoids Enhance the Retention of Transplanted Stem Cells and Promote Rapid Tissue Regeneration

**Figure 7:**
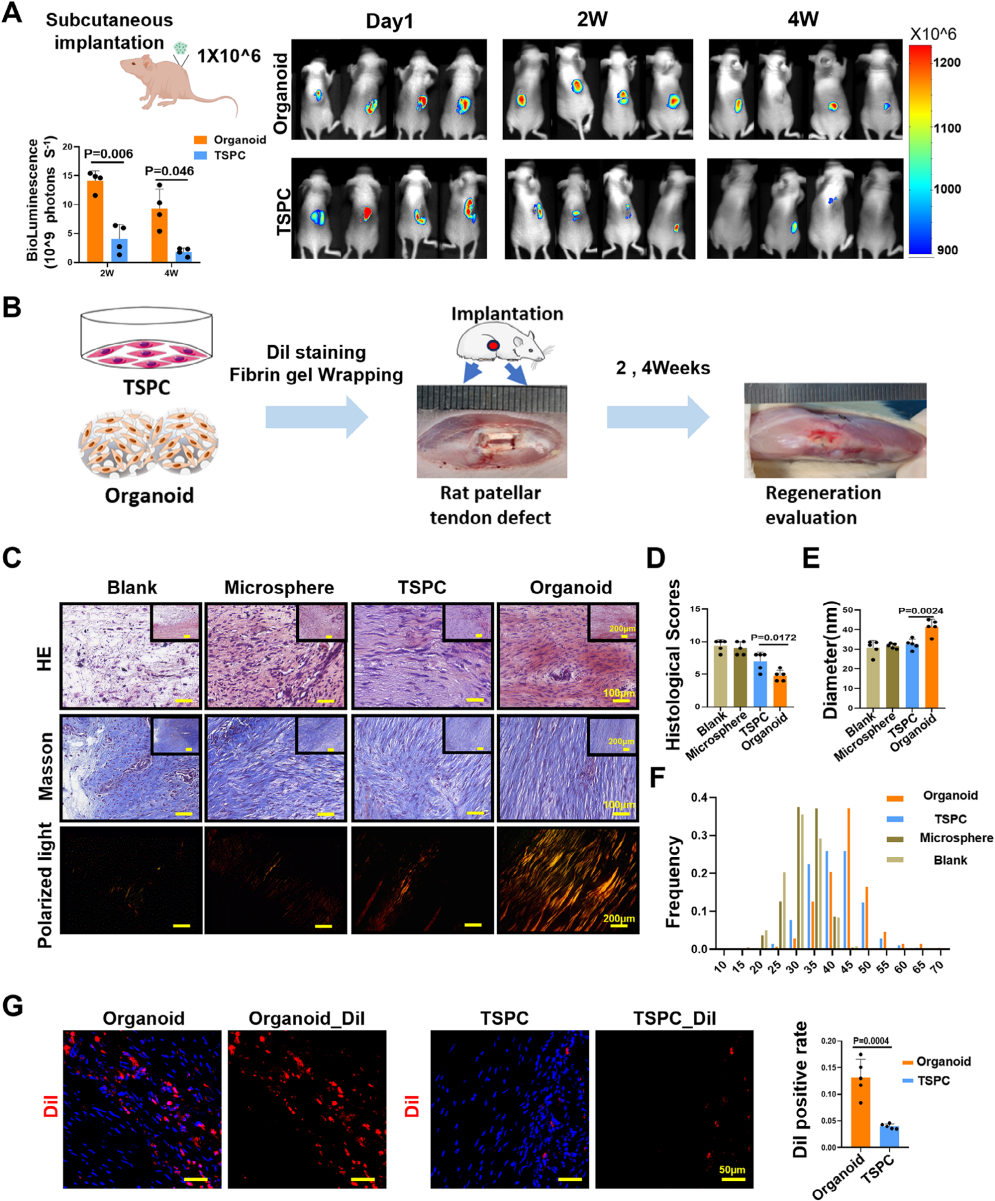
TSPCs organoid demonstrated an enhanced tendon regeneration capacity at 2 weeks after surgery. A) Fluorescence imaging of tendon organoids implanted in the back of nude mice (*n* = 4). B) Schematic illustration of implantation of organoid in patellar tendon defect model of SD rats. C) H&E, Masson, and polarized light images of the repaired rat patellar tendons at the 2 weeks post‐surgery. Scale bar: 100 µm (H&E and Masson), 200 µm (polar light). D) Histology scoring of the rat repaired tendons (*n*  =  5). E,F) TEM of cross sections and longitudinal sections of the repaired collagen fibrils. E) The average diameter and F) frequency of the collagen fibril diameters (*n*  =  5). G) Immunofluorescence staining and statistic analyze of DiI in repaired tendons 2 weeks after surgery.

To evaluate the efficacy of TSPCs organoids in boosting in vivo tendon regeneration, we transplanted the organoids into the dorsal region of nude mice to assess their stem cell retention efficiency. the tendon organoids significantly improved the viability and retention efficiency of stem cells. The organoid group exhibited a significantly enhanced stem cell retention efficiency, achieving a 3.44‐fold higher retention rate compared to the clinical‐grade biomaterial (fibrin gel) group at 2 weeks post‐transplantation, and a remarkable 5.10‐fold increase by 4 weeks (**Figure** **7**A). We then implanted these organoids into a rat patellar tendon repair model (Figure 7B). MSs group and TSPC group were regarded as control. Two weeks post‐implantation, the repaired tendons in the organoid group showed dense arrangement without evident inflammation or swelling. In contrast, the 2D TSPCs group and MSs groups, displayed loosely arranged, scattered collagen structures with rounded tenocytes, alongside significant indications of inflammation and swelling in blood vessels (Figure 7C). To quantify the maturity of the regenerated tendon, we used histological scores, which were notably improved in the organoid group compared to other groups (Figure 7D). Collagen, the principal component of tendons and a major constituent of the ECM, was further examined. Two weeks post‐surgery, TEM of the repaired tendon showed that collagen fibers in the organoid group were larger, denser, and better aligned when compared to the TSPCs and control groups (Figure S7A,B, Supporting Information and Figure 7E,F).

To investigate the differentiation fate of post‐implantation tendon organoids in injury area, various tenogenic markers were assessed. When compared to the TSPCs and other control groups, *MKX* and *NES* were expressed significantly higher in the organoids group (Figure S7C,D, Supporting Information). The organoid labeled with DiI were observed to have achieve higher survival by 3.285 times up to 13.14% in the tendon injury area, reflecting that organoid provided strong local residency (Figure 7G). In vivo experiments on animal models demonstrated that organoid have significantly enhanced abilities for tendon formation and tissue repair at the two‐week post surgery.

**Figure 8:**
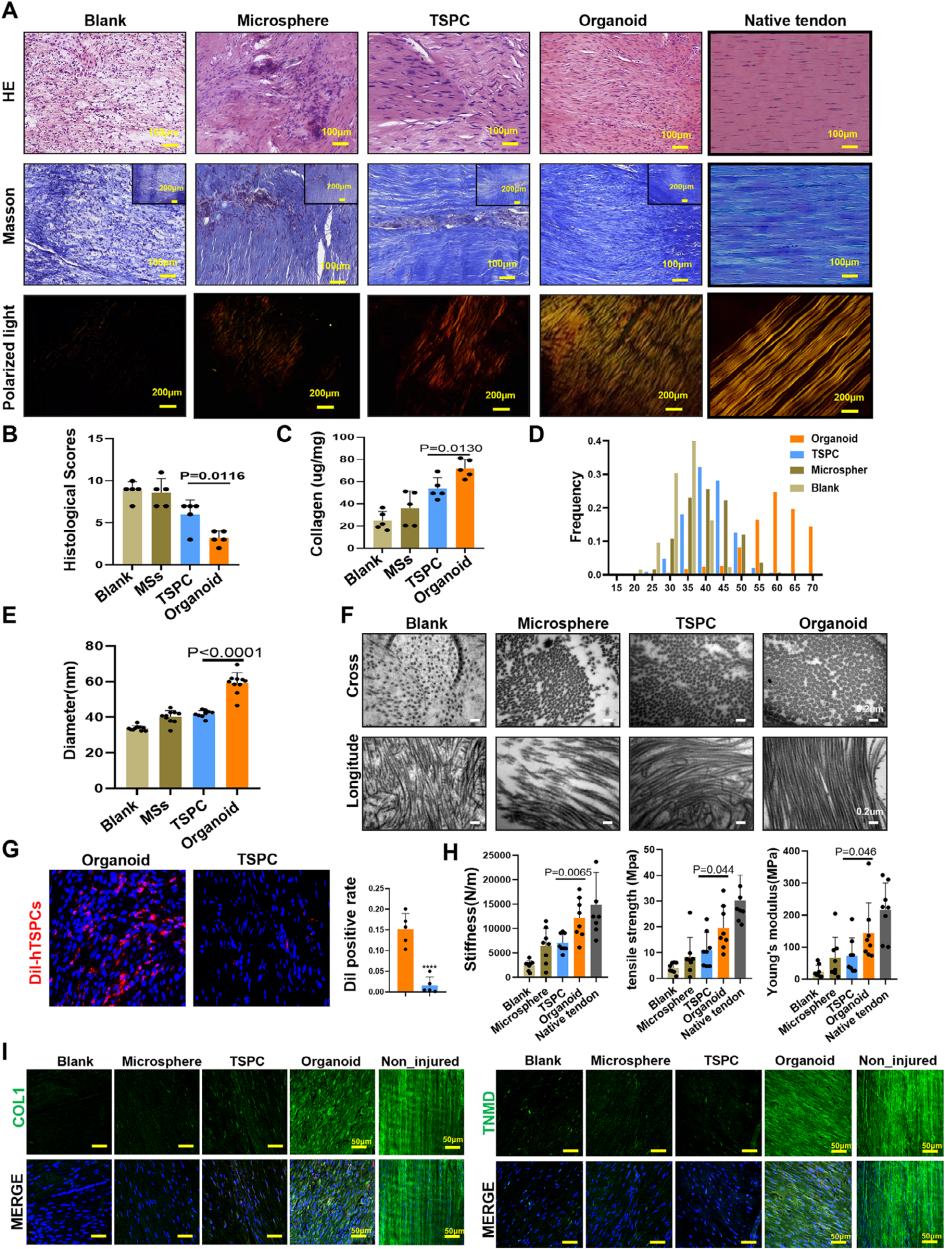
TSPCs organoid demonstrated an enhanced tendon regeneration capacity at 4 weeks after surgery. A) H&E, Masson, and polarized light images of the repaired rat patellar tendons at the 2 weeks post‐surgery. Scale bar: 100 µm (H&E and Masson), 200 µm (polar light). B) Histology scoring of the rat repaired tendons (*n*  =  5). C) Collagen content analysis of repaired tendons tissue (*n*  =  5). D–F) TEM of cross sections and longitudinal sections of the repaired collagen fibrils. Scale bars: 200 nm. G) Immunofluorescence staining for DII in the repaired regenerated tendon at 4 weeks after surgery. Scale bars: 50 µm. H) Mechanical properties (stiffness, tensile strength and Young's modulus) of the regenerated tendons at four weeks after surgery (*n*  =  8). I) Immunofluorescence staining for Col1 and TNMD in the repaired regenerated tendon at 4 weeks after surgery. Scale bars: 50 µm.

To further evaluate the prognosis of tendon injury repair, we assessed the regenerated tendons four weeks after surgery. Compared to the two‐week group, the four‐week organoid group presented a denser distribution of spindle‐shaped cells aligned with the tensile load direction, along with decreased inflammation, as confirmed by H＆E, Masson, and polarized light staining. Contrarily, the repaired tendons in the TSPCs group and control group exhibited multicellular structures, disorganized collagen fibers, as well as more diffuse inflammation and swelling (**Figure** **8**A). Histological scores also demonstrated statistically improved tendon quality in the organoid group, compared to others (Figure 8B). Furthermore, more collagen, indicative of more complete and mature regenerated tendons, was deposited in the organoid group than the others (Figure 8C). Analyses of the diameter, diameter range distribution, and angular distribution of collagen fibers also showed the organoid group had larger and more aligned collagen fibers by the 4th week (Figures 8D—F and S7B, Supporting Information). Four weeks after implantation, DiI labeled transplanted cells was also highly detected by 7.9 times up to 15% in regenerating tendons of organoid group. In addition, Dil positive cells in organoid group 4 weeks was further increased than 2 weeks indicating that the implanted TSPCs were still actively involved in the repair process, showcasing that organoids provide a suitable adhesive microenvironment (Figure 8G). Mechanical strength, crucial in evaluating the motor function of regenerated tendons, was superior in the organoid group as compared to the TSPCs group and the other control groups (Figure 8H and Figure S7C, Supporting Information). The organoid group exhibited significantly higher levels of force and stress at injury zone. Stress at failure was 1.77 times higher in the organoid group compared to the TSPC control groups, respectively. The expression of tendon‐specific markers COL1, TNMD, and NES were also higher in the organoid group compared to the TSPCs and control groups (Figure 8I and Figure S7A, Supporting Information).

In conclusion, the regenerated tendons in the organoid group demonstrated superior architecture and mechanical properties compared to the other groups, underlining the TSPCs enhanced tendon regeneration capabilities in an organoid microenvironment. The implantation of tendon organoids can indeed promote a more efficient tendon repair process when compared to the traditional method of directly implanting TSPCs.

## 3 Discussion

Stem cell organoid transplantation represents a promising frontier in regenerative medicine research.^[^ ^30^ ^]^ In this study, we successfully engineered functional, centimeter‐scale tendon organoids using a tailored 3D culture system. The tendon organoid exceeding 3 centimeters in vitro, demonstrated remarkable proliferative potential, enriched ECM components, and a longitudinally arranged fibrous structure under tenogenic conditions. At the single‐cell level, these organoids successfully achieved tendon lineage‐specific differentiation, showing significantly greater similarity to tendon‐resident TSPCs compared to 2D or scaffold‐only 3D cultures. When compared with embryonic and adult tendon tissues, the organoids retained the high proliferative capacity characteristic of embryonic tendon‐derived TSPCs. Upon integration into in vivo tendon defects, the organoids exhibited exceptional long‐term viability, achieving a 7.9‐fold higher retention rate of single cells, while promoting effective tendon regeneration. This innovative approach enabled rapid repair of tendon defects, with substantial functional recovery observed within four weeks. This work represents a novel and promising strategy for cultivating human‐scale organoids for regenerative applications. While iPSCs represent a broadly expandable and versatile stem cell source, their tendon differentiation requires complex, time‐consuming induction protocols and carries risks of tumorigenicity. Tendon stem/progenitor cells (TSPCs), by contrast, are tissue‐resident progenitors with inherent tenogenic commitment and epigenetic memory, enabling more direct, efficient, and safer tendon organoid formation. TSPCs also exhibit lower cellular heterogeneity and a well‐established safety profile, which supports reproducibility and clinical translation. Our optimized TSPC culture system further enhances rapid expansion and scalable generation of centimeter‐scale tendon organoids, underscoring practical advantages over iPSC‐based methods. Nevertheless, a recent study by Tsutsumi et al.^[^ ^31^ ^]^ demonstrated the feasibility of generating tendon‐like tissues from iPSCs, we recognize the value of iPSC‐derived models and intend to integrate these in future studies to broaden the clinical applicability of organoid engineering.

A critical challenge in generating human‐scale tendon grafts lies in the progressive loss of TSPC phenotypic traits and proliferative capacity during in vitro culture.^[^ ^32^, ^33^ ^]^ This decline, characterized by reduced expression of tendon‐specific transcription factors (e.g., *SCX, MKX, EGR1, NES*) and diminished tendon‐forming capabilities, significantly limits the clinical potential of the construction and treatment of organoids.^[^ ^22^, ^23^, ^34^ ^]^ Previous studies have shown that serum‐containing media can cause batch‐to‐batch variability and may not fully replicate the biochemical cues required for optimal stem cell function.^[^ ^18^ ^]^ To overcome this barrier, we employed a small‐molecule reprogramming approach to optimize the culture system, thereby preserving TSPC functionality. RNA sequencing revealed that the organoid group achieved a transcriptional profile closely resembling that of fetal tendon tissue, with enhanced states of proliferation, self‐renewal, and tendon‐specific lineage. These findings were further corroborated by in vitro studies, where the organoid group exhibited significantly increased expression of tendon markers (*MKX, EYA2, NES*) and anti‐aging markers (*LAMIN B1, Ki67)*, alongside a marked downregulation of aging markers (β‐gal) at later passages. These advancements underline the potential of our culture system not only to produce functional tendon organoids for therapeutic applications but also to serve as a robust platform for investigating tendon biology, enabling precision drug screening, and exploring regenerative medicine strategies at a translational scale.

In previous studies leveraging developmental engineering principles, both stem cell sheets and tissue engineering scaffold have achieved expansion of grafts.^[^ ^35^, ^36^ ^]^ That provides valuable insights for simulating organ development in vitro. However, there exist remarkable differences between the previous studies and the organoids in this study. The most significant difference lies in our achievement of both human tissue‐scale dimensions and molecular‐level tendon biomimicry. Not only due to the high‐proliferation culture system, but also because of the porous structure and support of the carrier, the implanted tendon stem cells in our research not only accomplished significant tendon formation and self‐assembly expansion via the interstitial spaces between adjacent carriers but also enabled the adequate interchange of nutrients in the core area of the microtissues. These features enabled the formation of fibrous structures characteristic of native tendons, demonstrating the critical importance of both the physical and chemical microenvironment in promoting tissue development. In native tendon development, mechanical stimulation serves as a critical physiological cue that drives the nuclear translocation of key transcription factors and promotes extracellular matrix remodeling and tissue maturation. While our current culture system successfully simulates biochemical cues essential for tendon development, it does not yet incorporate mechanical stimuli. In future studies, we aim to integrate physiologically relevant mechanical loading into our platform to further enhance the maturation and functionality of tendon organoids. When the core region of organoid was directly observed using transmission electron microscopy, it was revealed that the collagen diameter and arrangement in organoid group were remarkably enhanced in comparison with the cell sheets induced for tendon formation. Our results also demonstrated that the formed tendon microtissues exhibited high expression of ECM components such as COL1 and COL3, with a substantial number of cells and good proliferative function in the core region under light sheet microscopy. In line with this, four weeks following implantation in the injured area, the retention rate of stem cells in the TSPC group was markedly lower than that in the organoid group. In addition, while our results demonstrated excellent organoid viability and regenerative function in vivo, the issue of immunogenicity remains critical for clinical translation. To preliminarily address this, we performed immunofluorescence staining of immune cell markers (CD3, CD8, CD56, and CD68) on grafts retrieved two weeks post‐implantation, which revealed no significant immune cell infiltration. This likely reflects the low immunogenicity of TSPCs, a mesenchymal stem cell (MSC) subtype that has already been widely applied in clinical settings. These results support the potential feasibility of allogeneic or autologous organoid transplantation with minimal immune rejection. Regarding scalability, our self‐assembling platform enables organoids to spontaneously expand to centimeter‐scale dimensions within two weeks, offering a promising strategy for producing clinically relevant grafts. Nonetheless, further work will be needed to adapt the current system to GMP‐compatible conditions and assess its robustness in larger‐scale or automated production pipelines.

In summary, by optimizing both the physical and chemical microenvironments, we have successfully constructed tendon organoids at human tissue scale. The high tissue fidelity and regenerative capacity of our organoids not only enables to s promote tendon repair effectively but also positions them as promising platforms for precision drug screening and toxicity evaluation for tendon injuries and tendinopathies in the future. However, challenges such as vascularization and neural innervation remain critical for ensuring the long‐term survival and functional integration of these constructs post‐transplantation. Addressing these aspects will be a key focus of future research.

## 4 Conclusion

In conclusion, this study successfully constructed centimeter‐scale tendon organoids for the first time, which excelled at promoting proliferation, differentiation ability and enhanced rejuvenation characteristics of TSPCs in vitro. By effectively balancing the proliferation and induction of differentiation in TSPCs, this approach not only facilitates the expansion of parenchymal cells but also induces the deposition of extracellular matrix, and finally promotes the self‐assembly of organoids into microtissues. The organoids application in vivo has demonstrated significant improvements in post‐transplantation retention, and the regeneration of injured tendons structurally and functionally. This innovative approach heralds promising potential for future implications in the field of tendon tissue engineering and regeneration.

## 5 Experimental Section

### Isolation and Culture of Human TSPCs

Previously established methods were employed to isolate and purify TSPCs.^[^ ^15^, ^22^ ^]^ All pertinent procedures involving the acquisition, isolation, and culture of TSPCs received ethical approval from the Ethics Committee of the Second Affiliated Hospital, School of Medicine, Zhejiang University (I2019001166). TSPCs were isolated from semitendinosus tendon tissues collected during anterior cruciate ligament (ACL) reconstruction surgeries from three independent donors (Male, 43 years old; Female, 31 years old; Male, 21 years old). All donors were free of infectious or systemic diseases. Tendon tissues were divided into minute portions and digested under an environment of 37 °C for a duration of 1 h, in a low glucose Dulbecco's Modified Eagle Medium (L‐DMEM) loaded with 0.20% Type I collagenase. Isolated cellular suspensions were then cultured in growth medium (comprising L‐DMEM, 10.00% Fetal Bovine Serum (FBS), and 1% Penicillin Streptomycin Solution (PS)) under conditions of 5% CO2, at 37 °C to form colonies for a period between 8 and 10 d. Upon attaining 80–90% confluence, the TSPCs were passaged. For the purposes of this study, TSPCs stemming from passages 3 through 6 were utilized.

### Organoid Culture

TSPCs were isolated and cultured from semitendinosus tendon tissues as described in Section 2.1. The concentration of the TSPCs suspension was adjusted to a range between 2–3 × 10 ^ 5 cells mL^−1^. Following this adjustment, a measure of 200 µL TSPCs suspension was introduced to a 20 mg quantity of 3D microcarrier (Cytoniche, Beijing, China) in a low‐adhesion six‐well plate, and incubated for 4 h at 37 °C under 5% CO_2_ to allow cell attachment on 3D microcarrier. After attachment, 2 mL of serum‐free tendon‐specific culture medium was gently added to each well. This medium was based on a tendon‐specific medium containing key growth factors essential for tendon lineage induction (the serum‐free medium is supplemented with F12/DMEM, fibroblast growth factor 2(FGF‐2, 20 ng mL^−1^, peprotech, USA), heparin (2 µg mL^−1^, Selleck, USA), vitamin C (80 µg mL^−1^, sigma, Germany), transferrin (15 µg mL^−1^, sigma, Germany), insulin (5 µg mL^−1^, sigma, Germany), progesterone (10 ng mL^−1^, Selleck, USA), putrescine (10 µg mL^−1^, sigma, Germany) and sodium selenite (10 ng mL^−1^, Gibco, USA)—mixed in any order at 0–37 °C.). The medium was refreshed every 2–3 d. Cultures were maintained in a humidified incubator at 37 °C with 5% CO_2_ for up to 28 d. Samples were collected at defined time points (e.g., Days 7, 14, and 28) for further analysis. Unless otherwise specified, all organoid experiments were performed using TSPCs from passages 3–6.

### Evaluation of Cell Viability and Proliferation

To assess cell proliferation capabilities, TSPCs cultured in 3D were separated from 3D microcarriers and treated with 3D FloTrix Digestive solution (Cytoniche, Beijing, China) to digest the microcarriers for 15–20 min. This was followed by digestion with 0.05% trypsin (Gibco) for an additional 2–3 min to create a single‐cell suspension. Cell census was conducted on the 1st, 3rd, 5th, 7th, 10th, and 14th days using a countstar, and the cell proliferation rate was calculated as the ratio of the number of cells harvested to the initial seeding count. A fluorescence microscope (Nikon) was used to investigate the live/dead cell staining status of TSPCs in the 3D microenvironment after 1, 3, 5, and 7 d. Additionally, the actin filaments in the cytoskeleton were stained with TRITC‐conjugated phalloidin (Merck), and nucleus were stained with 4′, 6‐diamino‐2‐phenylindole (DAPI, Beyond), before being observed with a confocal microscope (Olympus). Drawing from previous studies,^[^ ^21^, ^26^, ^27^ ^]^ the cell area (*A*c) and cell volume (*V*c) were calculated using Image J software. The nucleocytoplasmic ratio was determined by calculating the ratio of the nuclear volume to cytoplasm volume. Sphericity was calculated as (π ^1/3^ (6Vc) ^2/3^)/Ac.

### Assessment of Multidirectional Differentiation Capacity

The ability of TSPCs to differentiate into osteogenic, adipogenic, and chondrogenic lineages was evaluated in both 3D microcarrier group and 2D culture group, as previously described.^[^ ^7^ ^]^ Before differentiation, TSPCs were cultured in either 2D or 3D microenvironments for a duration of 4 d. Afterwards, TSPCs were digested from 3D microcarriers or 2D Petri dishes for ensuing experimental procedures. To facilitate osteogenic differentiation, TSPCs were cultured in high‐glucose DMEM (H‐DMEM) for 7 d, supplemented with 10 mm β‐glycerophosphate, 50 µg mL^−1^ vitamin C, and 10 ^ ‐8 m dexamethasone (all procured from Sigma Aldrich). The osteogenic differentiation of TSPCs was evaluated using Alkaline Phosphatase Staining (ALP, Beyotime), with cell counts determined after DAPI normalization. Chondrogenesis differentiation required 20 µL 5 ×10 ^ 5 cell suspensions to be pipetted into a single well of a 24 well plate, housed in H‐DMEM, with the supplementary addition of 1% ITS, 1 mm sodium pyruvate, 50µg mL^−1^ vitamin C, 10—7 m dexamethasone, and 10 ng mL^−1^ TGF‐β3. After 14 d, the microcapsules were stained with Alcian blue to assess their chondrogenic capability. For adipogenic differentiation, cells were cultured using the MesenCult Adipogenic Differentiation Kit (Human) (StemCell Technologies) for 21 d. The evaluation of adipogenesis was performed using Oil Red O staining.

### Flow Cytometry Analysis

TSPCs procured from both 2D and organoid groups were prepared by fixing them with 1% (w/v) polyformaldehyde (PFA). This was followed by an incubation process of 5 × 10 ^ 5 cells with 1 µg of either APC or PE conjugated antibodies, which took place on ice for 30 minutes. Post‐incubation, separate staining procedures were conducted for CD105 and CD44. Detailed information of antibodies used is provided in Table S1 (Supporting Information). After being washed with phosphate buffered saline (PBS), samples were analyzed using Cytomics FC500 Flow Cytometry (Beckman).

### Immunofluorescence Staining

After 1–14 d of culturing in a 3D microenvironment, the cells were first fixed in 4% (w/v) PFA for 30 min. Then, they were permeabilized with 1% (v/v) Triton X‐100 in PBS for 10 min and subsequently blocked with 1% BSA (w/v) for another 30 min. The samples were then incubated with primary antibodies overnight at a temperature of 4 °C. The primary antibodies used were: anti‐collagen I, anti‐Lamin B1, anti‐TNMD, anti‐NESTIN, anti‐MKX, anti‐EGR1 anti‐COL3, EDU, anti‐KI67, and anti‐PMTOR. After the incubation period, samples were washed three times with PBS and subsequently incubated with goat anti‐rabbit or mouse secondary antibodies conjugated with Alexa Fluor 488 or 546 fluorescent dye (Invitrogen). DAPI was used to stain the nuclei. Through the Z‐stack function, 3D immunofluorescence images were procured and composed into a singular image. These fluorescence images were then obtained using a confocal microscope (Olympus). 3D renderings were generated using Imaris 9.6.1 software.

### Scanning Electron Microscope (SEM)

After the sample was treated with a gold coating to augment its conductivity, we employed SEM (FEI, Nova Nano 450) to closely examine the morphology of both the microstructure and the TSPCs, which had been cultured in 3D microcarriers for 1–7 d. This process was conducted under an accelerated voltage of 3kV. ROI manager and Image J software were used to analyze the SEM images.

### Bulk RNA‐Sequencing, Single‐Cell RNA‐Sequencing (scRNA‐Seq) and QRT‐PCR

For bulk RNA sequencing, TSPCs from a single donor were divided into two groups and cultured either on 2D dishes or on 3D microcarriers in tendon‐specific induction medium. After 3 d of culture, cells were harvested using TRIzol reagent (Takara, 9109) for total RNA extraction. Following quality assessment, high‐quality RNA was used for cDNA library construction (Biozeron, Shanghai, China) and subsequent sequencing.

For scRNA‐seq, adult tendon, fetal tendon, 2D‐cultured, 3D microcarrier‐cultured, and organoid‐cultured TSPCs were collected for analysis. Fetal Achilles tendon sample (*n* = 1) were obtained at 12 weeks of gestation, with the fetal samples dated from the last menstrual period (LMP). The acquisition and sequencing of the samples were approved by the Ethics Committee of the Fourth Affiliated Hospital of Zhejiang University (K2021065), with informed consent obtained from all participants. Adult tendon tissue (*n* = 1) was collected during anterior cruciate ligament (ACL) reconstruction from a female donor (46 years old), with informed consent and approval from the Ethics Committee of the Second Affiliated Hospital (I2019001166). Tissue samples were first minced and digested with 0.2% collagenase I (Gibco) diluted in low‐glucose DMEM (Gibco) at 37 °C for 2 h. For 3D and organoid samples, digestion was performed with microcarrier digestion, followed by filtration through a 70 µm mesh to remove undigested cell clumps. The resulting cell suspension was then adjusted to a concentration of 5 × 10^5 cells mL^−1^ for library preparation. Single‐cell capture, RNA extraction, and cDNA synthesis were carried out following the Fluidigm protocol for single‐cell RNA sequencing. The qualified libraries were then sequenced on the Illumina HiSeq X Ten platform. For data analysis: the raw counts were normalized using Seurat (version 4.3.0.1). Genes significantly enriched in each cell cluster were identified using Seurat's default algorithm. Cell subpopulations were annotated based on marker genes and their matching with standard markers. Functional annotation of the generated marker gene list relative to Gene Ontology (GO) terms was performed using Metascape.^[^ ^37^ ^]^ Trajectory analysis was conducted using Monocle (version 2.10.1).

For qRT‐PCR, TSPCs were we collected from 2D and 3D microcarriers using Trizol to yield RNA, which was subsequently reverse transcribed into cDNA. Using SYBR Green Mix (Takara, RR420A) for qRT‐PCR on a Light Cycler 480 system (Roche). Details regarding the primers used are provided in Table S2 (Supporting Information).

### Assessing the Stem Cell Retention Tendon Formation and Regeneration Ability in Animal Experiments

Stem‐cell retention was first quantified in nude mice; the same organoid constructs were then evaluated in a Sprague–Dawley rat patellar‐tendon window to determine their capacity to enhance structural and biomechanical repair. To evaluate the in vivo retention capacity of tendon organoids, organoids or 2D‐cultured hTSPC sheets were transplanted into a nude mouse subcutaneous model, following previously described protocols.^[^ ^22^ ^]^ After anesthesia, a 2 cm subcutaneous incision was made on the dorsal side of the mice. Tendon organoids or cell sheets (prelabeled with the fluorescent dye DiI, Invitrogen, C7000) were sutured beneath the dorsal skin. Postoperatively, in vivo retention of the transplanted constructs was monitored using a small animal live imaging system (Biospace Optima, France). To assess the tendon regeneration capacity of TSPCs cultured in 2D or 3D animal models, we utilized female adult Sprague Dawley rats weighing 200—220 g. All guidelines for animal care and use, as stated by relevant institutional and national bodies, were strictly adhered to. This research protocol has received approval from the Animal Protection and Utilization Committee of Zhejiang University (ZJU20220230). 48 h before implantation, TSPCs were labeled with DiI to track of the survival rate of transplanted cells at the injury site. The cells were incubated in L‐DMEM for 24 h, after which they were wrap with fibrin gel. A dosage of 150 mg kg^−1^ of cyclophosphamide was administered 24 h before surgery. After ensuring the rats were anesthetized, a notch wound (1.5 mm in width and 4 mm in length) was created on the patellar tendon of each rat by following previously detailed procedures. The fibrin gel, carrying 20 µL cells, was then employed to suture the patellar tendon defect. In the microtissue group, 5×10^5 TSPCs cultured in 3D microcarriers within 20 µL fibrin gel were used to treat the tendon defect. Likewise, 5×10^5 TSPCs cultured under 2D environments in 20 µL fibrin gel were used for the 2D groups while 20 µL fibrin gel alone was used for treating tendon defects in the blank control group (Ctrl). For the simple microcarrier control group, 20 µg microcarrier was used to treat the tendon defect. Following the treatment, the wounds were sutured, and the rats were allowed to move around freely. At the 2‐week and 4‐week post‐surgery marks, the repaired patellar tendons were assessed through histology, transmission electron microscope (TEM), magnetic resonance imaging (MRI), mechanical testing, and collagen content determination. Every experiment was conducted with 5 independent samples per group.

### Transmission Electron Microscope (TEM)

As standard TEM procedures, cell slices cultured in vitro or regenerated tendon samples from animal models were prepared to determine collagen fibril diameter and arrangement. These samples underwent fixation in 2.5% glutaraldehyde at 4 °C overnight, followed by thrice washing in PBS. They were additionally fixed with 1% OsO4 in PBS for 1.5 h, succeeded by another three rounds of washing with PBS. Subsequently, samples were dehydrated using graded ethanol (30%, 50%, 70%, 80%) and graded acetone (90%, 95%, 100%), each for 15 min. Thereafter, the specimens were immersed in a mixture of absolute acetone and Spurr resin at 1:1 for 1 h, followed by 1:3 for 3 h, and then left in a Spurr resin mixture overnight. After placing samples in fresh Spurr resin and heating at 70 °C for a minimum of 9 h, they were sectioned using an ultra‐microtome (LEICAEMUC7) and then stained with uranyl acetate and alkaline lead citrate for 5–10 min, respectively. The final imaging process was performed using a Hitachi H‐7650 transmission electron microscope. To ensure accurate representation of fiber diameter and distribution, five samples were selected per group, with each sample yielding 2–4 images. Approximately 500 collagen fibrils were measured from each tissue sample. As mentioned in previous studies,^[^ ^30^, ^37^ ^]^ the direction distribution was automatically calculated by ImageJ/OrientationJ.

### Evaluation of Mechanical Properties of Regenerated Tendons

The mechanical properties were examined using the Instron tensile/compression system (model 5543, Instron, Canton, MA), as previously described.^[^ ^10^ ^]^ The hind limbs from rats used in the prior animal model experiments were harvested, ensuring the patellar tendon was preserved while removing other soft tissues around the knee. The cross‐sectional area of the patellar tendon was measured and recorded and the femur‐patella‐tendon‐tibia complex (FPTC) was fixed onto a customized clamp. An initial pre‐treatment was conducted on each FPTC with a preload of 0.1N, applying a cyclic elongation ranging between 0 and 0.5 mm over 20 cycles at a rate of 5 mm min^−1^. A load failure test was then conducted at a tensile speed of 5 mm min^−1^. The assessment of the mechanical properties of the regenerated patellar tendon was based on its stiffness (N m^−1^), destructive force (N), the energy absorption at failure (Joules), modulus (Megapascals), stiffness per unit cross‐sectional area (Mpa), and stress at failure (Mpa).

### Statistical Analysis

The quantitative data are presented as mean ± standard deviation (SD). For comparisons between two independent groups, unpaired two‐tailed Student's t‐test was used. For comparisons among multiple groups, one‐way ANOVA followed by Tukey's post hoc test was performed. In these multiple‐group comparisons, we further conducted pairwise evaluations to specifically assess the improvements of the organoid group relative to control group (Figures 7D,E and 8B,C,E,H). Any value where *P* < 0.05 was deemed to be statistically significant.

## Conflict of Interest

The authors declare no conflict of interest.

## Author Contributions

T.F., H.Z., and Y.X. contributed equally to this work. T.F.: conceptualization, methodology, validation, investigation, formal analysis, data curation, writing, project administration, visualization and writing—review & editing. H.Z.: conceptualization, methodology, investigation and writing—review & editing. Y.X.: methodology, software, formal analysis and visualization. X.L.: investigation, resources and data curation. X.L.: methodology, investigation and resources. Z.W.: investigation and resources. Y.X.: investigation and resources. X.X.: investigation. Z.W.: investigation. T.L.: formal analysis. R.L.: formal analysis. W.S.: resources. B.W.: resources. Y.D.: resources, and supervision. X.C.: conceptualization, validation, writing—review & editing, supervision and funding acquisition. Z.Y.: conceptualization, validation, writing—review & editing, supervision, project administration and funding acquisition.

## Supporting information



Supporting Information

## Data Availability

The raw/ processed data necessary to replicate these findings cannot be shared at the moment, as this data is also being utilized for an ongoing study.
