# Supplementary material for: Centimeter‐Scale Self‐Assembling Tendon Organoids Drive Tissue Regeneration
Source: Adv Sci (Weinh). 2025 Aug 29;12(43):e09453. doi: 10.1002/advs.202509453 (PMC12631886; doi:10.1002/advs.202509453)
Supplement: Supplementary file 1 — Supporting Information [file ADVS-12-e09453-s001.docx]

Centimeter-scale self-assembling tendon organoids drive tissue regeneration

Tianshun Fang^1,2#^, Hong Zhang^7#^, Yuanhao Xie^1,3,5#^, Xiongfeng Li^8^, Xi Liu^7^, Zicheng Wang^7^, Yiwen Xue^2^, Xiaohui Xia^2^, Zetao Wang^1,2,3^, Tingyun Lei^1,2,3^, Ruifu Lin^1,2,3^, Weiliang Shen^1,5^, Bingbing Wu^2,9^, Yishan Chen^2,4^, Yanan Du^6^, Xiao Chen^2,3,5*^, Zi Yin^1,2*^

^1^ Department of Orthopedic Surgery of Sir Run Run Shaw Hospital, and Liangzhu Laboratory, Zhejiang University School of Medicine.

^2^ Dr. Li Dak Sum & Yip Yio Chin Center for Stem Cells and Regenerative Medicine, Zhejiang University School of Medicine, Hangzhou, PR China.

^3^ Key Laboratory of Motor System Disease Research and Precision Therapy of Zhejiang Province, Hangzhou, Zhejiang Province, China

^4^ Zhejiang University-University of Edinburgh Institute, Zhejiang University International Campus, Haining, 314400, China.

^5^ Department of Sports Medicine & Orthopedic Surgery, the Second Affiliated Hospital, Zhejiang University School of Medicine, Hangzhou, China

^6^ Department of Biomedical Engineering, School of Medicine, Tsinghua-Peking Center for Life Sciences, Tsinghua University, Beijing, 100084, China.

^7^ Center for Rehabilitation Medicine, Rehabilitation & Sports Medicine Research Institute of Zhejiang Province, Department of Rehabilitation Medicine, Zhejiang Provincial People's Hospital, Affiliated People's Hospital, Hangzhou Medical College, Hangzhou, Zhejiang, China, Hangzhou, 100084, China.

^8^ Huzhou Central Hospital, Zhejiang University School of Medicine, Huzhou, Zhejiang, China.

^9^International Institutes of Medicine, The 4th Affiliated Hospital of Zhejiang University School of Medicine, Hangzhou, Zhejiang, China

**^#^** These authors contributed equally

^*^Corresponding author:

Zi Yin, Department of Orthopedic Surgery of Sir Run Run Shaw Hospital, and Liangzhu Laboratory, Zhejiang University School of Medicine, Hangzhou, China. e-mail: [yinzi@zju.edu.cn](mailto:yinzi@zju.edu.cn)

**Supplementary Materials**


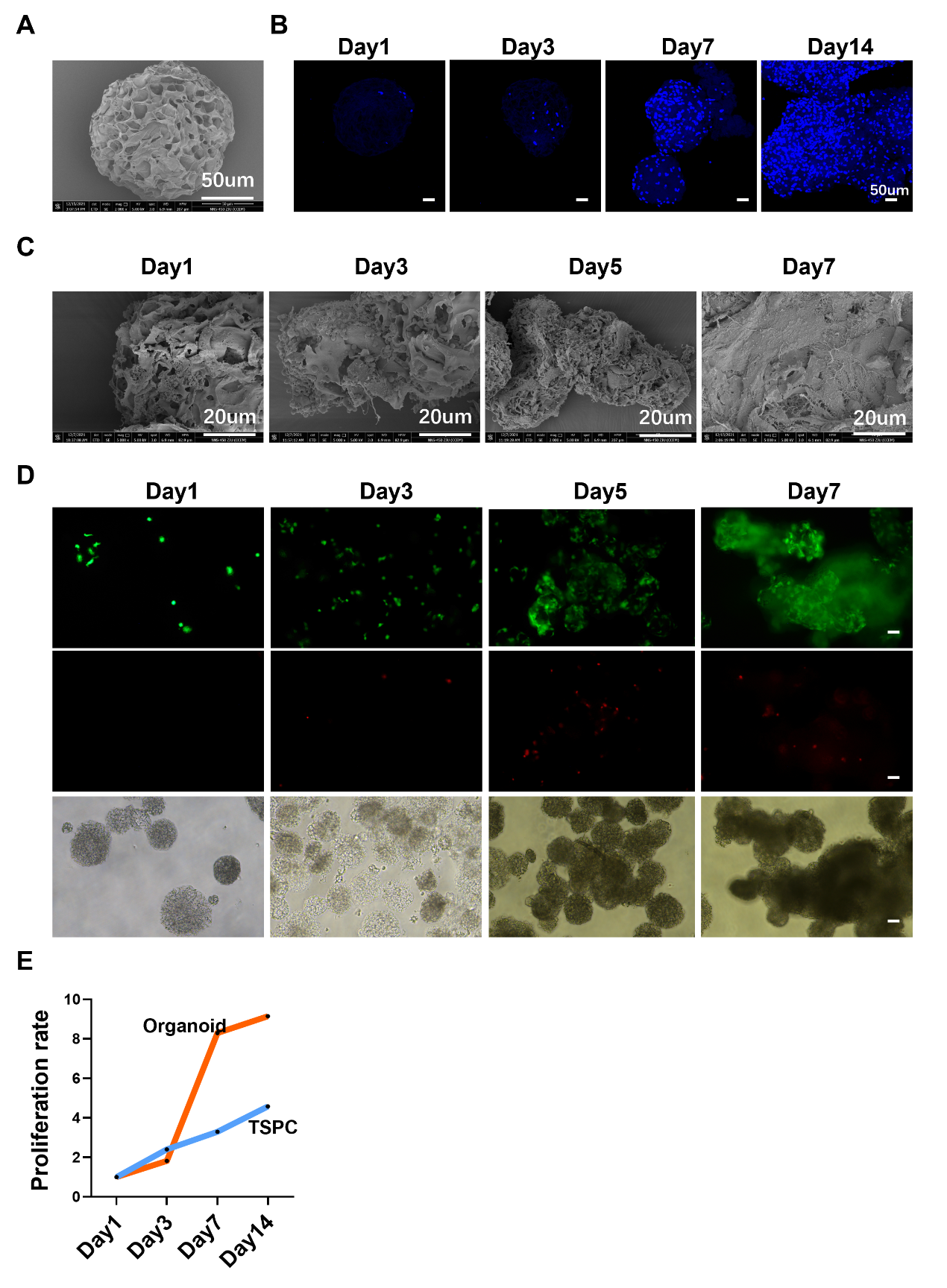


Fig. S1. Scale expansion of hTSPCs in tendon organoids. (A) SEM images exhibiting the porous ultrastructure of the 3D microcarriers. Scale bars: 50 μm. (B) DAPI staining exhibiting hTSPCs Proliferated well on the microcarriers. Scale bars: 500 μm. (C) Scanning electron microscopy (SEM) images exhibit the self-assembly and proliferation morphology of hTSPCs in the organoid group at 1,3,5 and 7 days. Scale bars: 20 μm, 50 μm. (D)Live/dead cell staining was conducted to demonstrated the good cell viability of hTSPCs in the organoid group at 1,3, 5 and 7days (n=3). (E). On-Scaffold CCK8 Assay for Assessing Organoid Proliferation Without Scaffold Digestion.


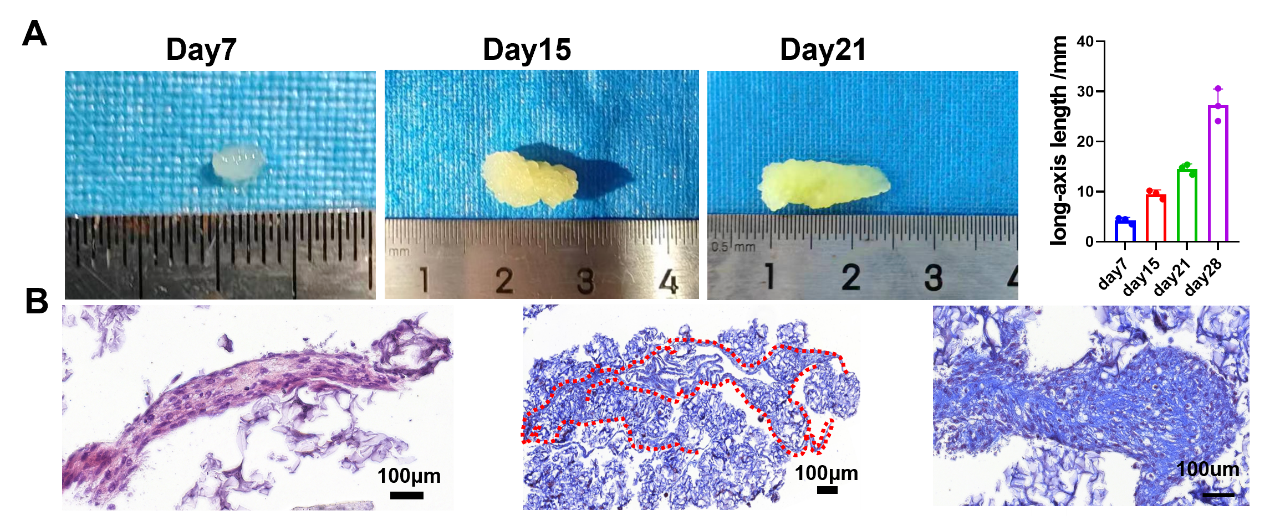


Fig. S2. Tendon organoids showed spontaneous aggregation into macroscopical microstructures in vitro. (A) Macroscopic view of tendon microtissue at 7, 15, and 21 days in vitro. (B) Images of tendon microtissue in vitro by HE staining and masson staining.


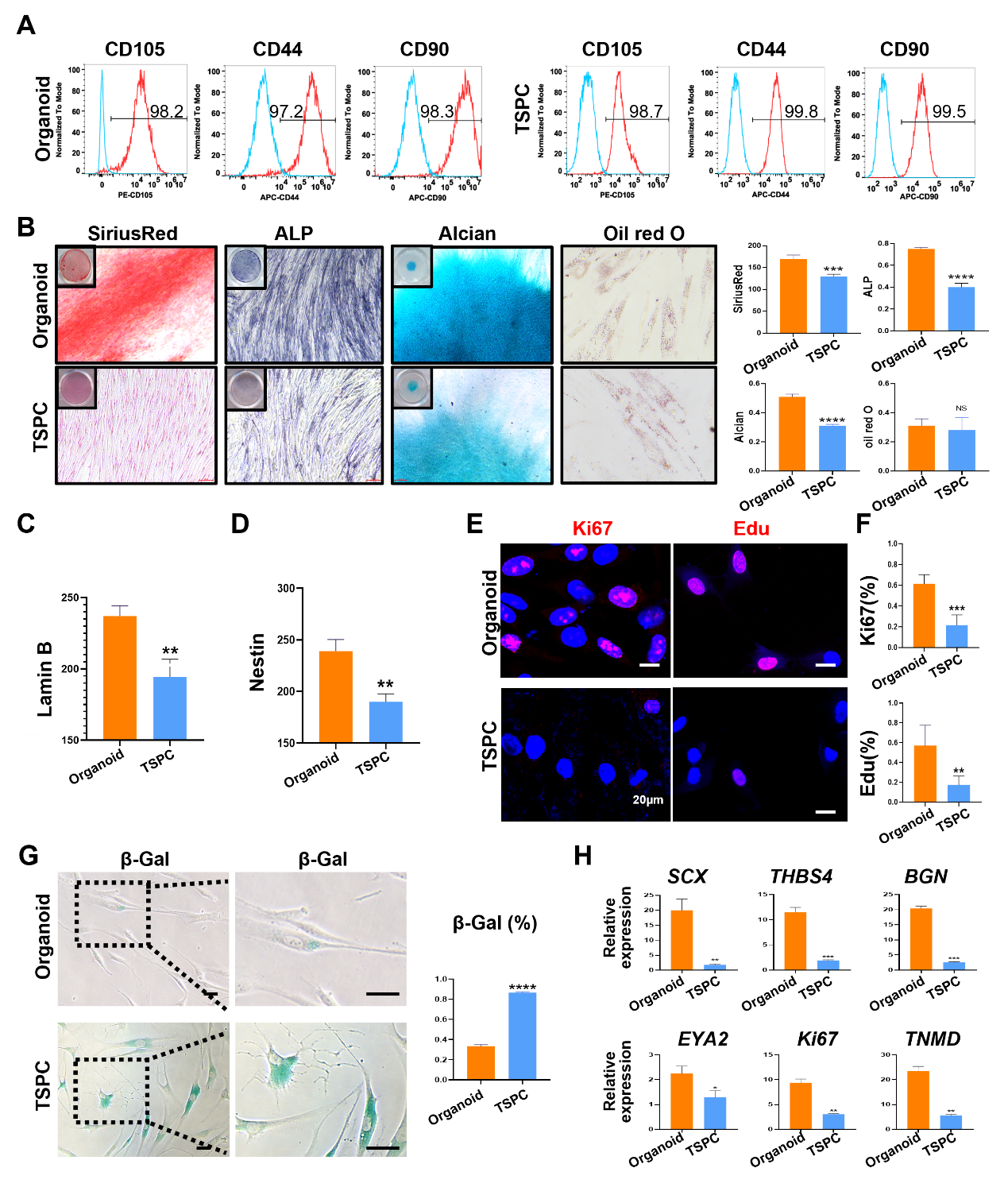


Fig.S3. Tendon Organoids Exhibit Multipotency and Anti-senescence Characteristics. (A) Flow cytometry analysis of hTSPCs in the organoid and control groups at 7 days. (B) Representative assessment of osteogenesis ability after induction for 7 days, ARS and ALP staining. Assessment of chondrogenesis ability after induction for 14 days. Alcian Blue staining. Assessment of adipogenesis ability of hTSPCs after induction for 21 days, Oil Red O staining. Scale bars: 50 μm. (C-F) Fluorescence microscopy images of hTSPCs staining Lamin B, Nestin, ki67 and EDU cultured in organoid and control at P8 day 4 in vitro. (G)Aging related β-gal staining of tendon organoid without induction at P8 day 4 in vitro, and the positive rate of βGal. (H) PCR analyses of the *SCX, THBS4, BGN, EYA2, Ki67*and*TNMD*of hTSPCs after being cultured in the organoid and control for 4 days (n = 3).


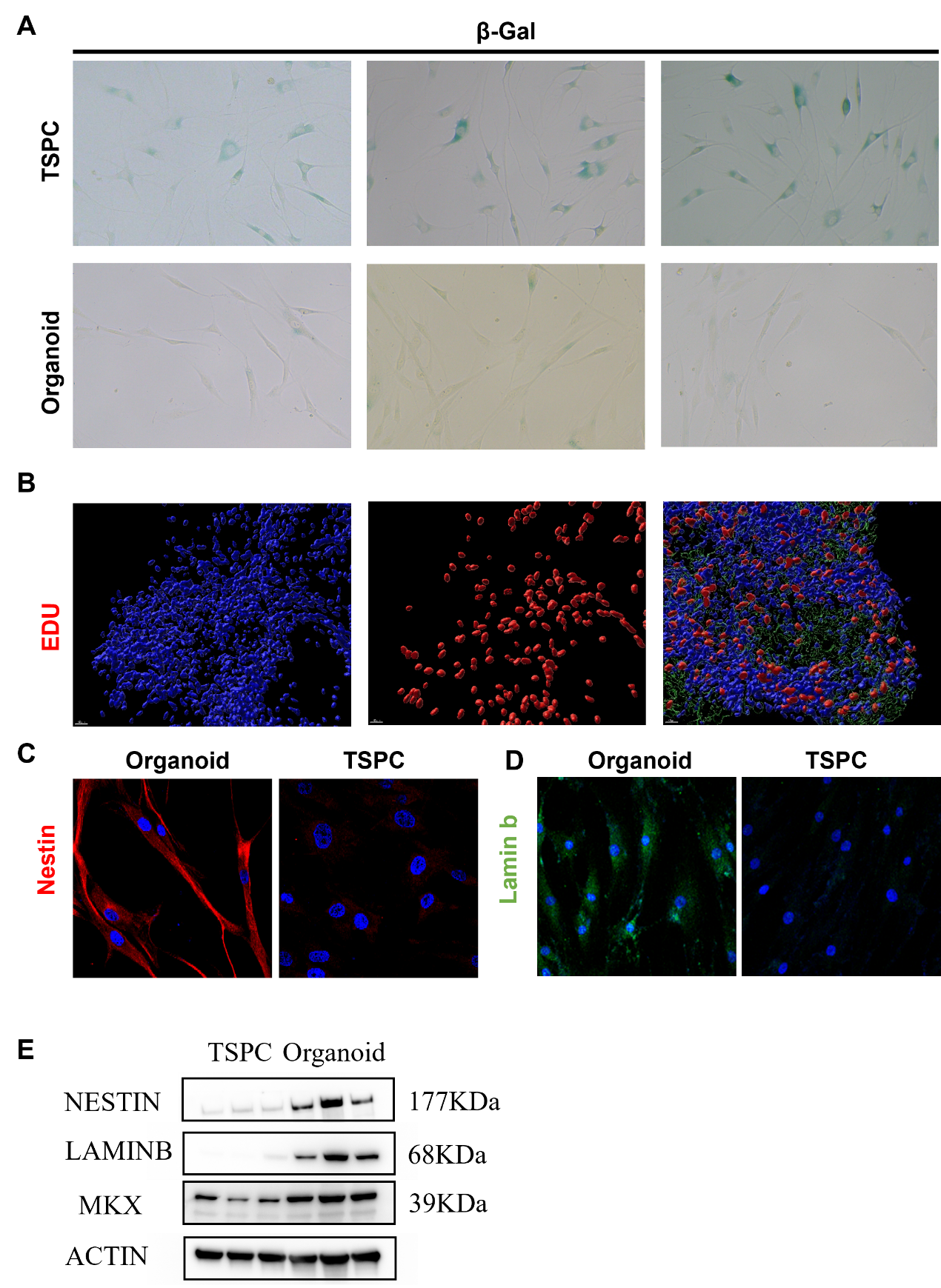


Fig. S4. Tendon Organoids Exhibit tenogenic capacity and Anti-senescence Characteristics. (A) Aging related βgal staining of tendon organoid without induction at P8 day 4 in vitro. (B) Edu staining of tendon organoid at P8 day 4 in vitro. (C) NES and LAMINB fluorescence staining of organoids. (D) Western Blot Analysis of Key Tenogenic Markers (MKX), Rejuvenation Marker (LAMIN B), and TSPC Marker (NESTIN)


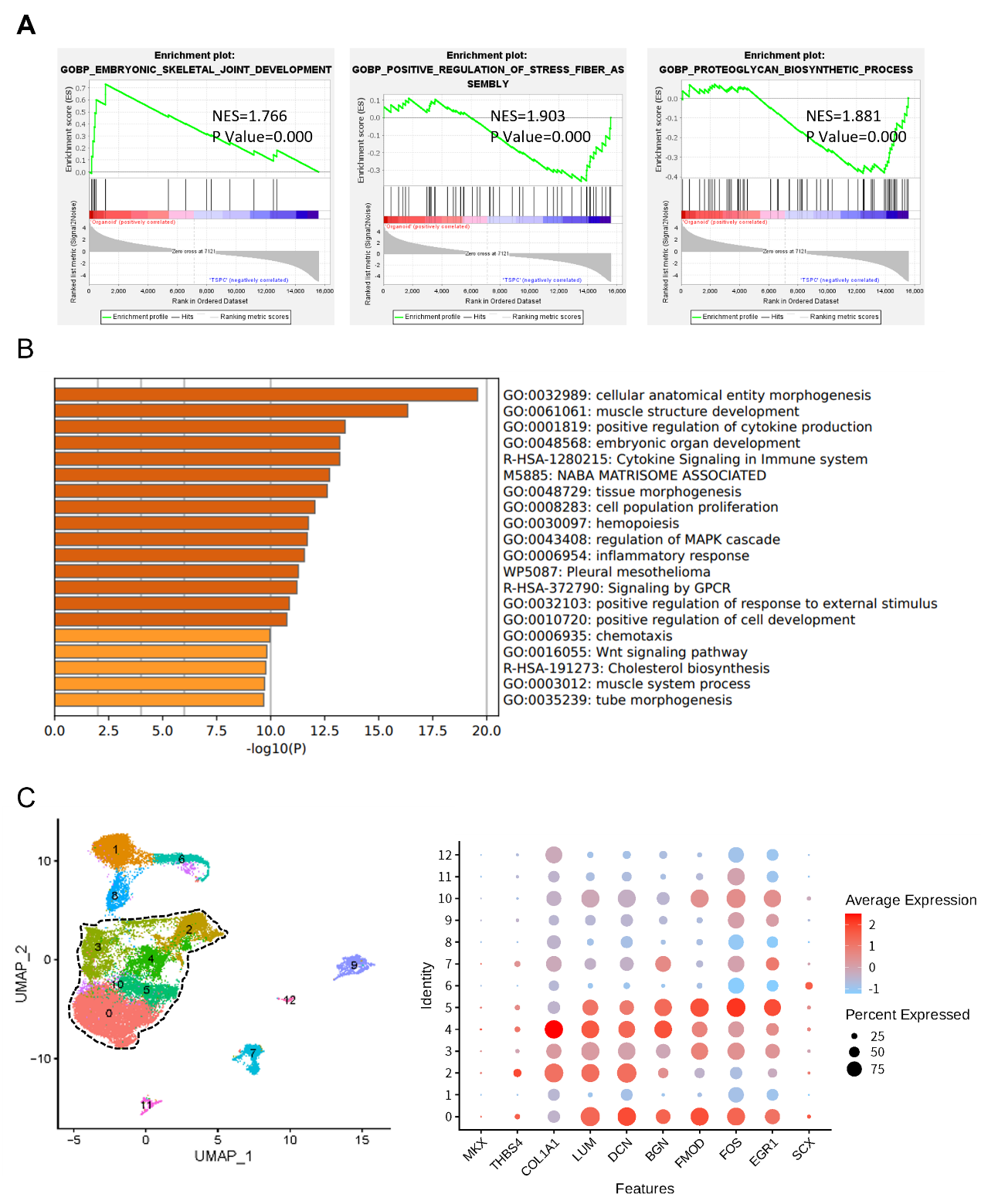


Fig. S5. Tendon organoids showed more excellent tendon generating ability and youth characteristics at the transcriptome level. (A) Gene Set Enrichment Analysis (GSEA) of the tendon related genes. NES = normalized enrichment score; FDR = false discovery rate. (B) Analysis of top20 enriched biological processes (GO) in organoid group compared with control; (C) Human tendon samples were first clustered based on key tendon markers (SCX, EGR1, FOS, FMOD, BGN, DCN, LUM, COL1A1, THBS4, MKX) to identify tendon-associated subpopulations


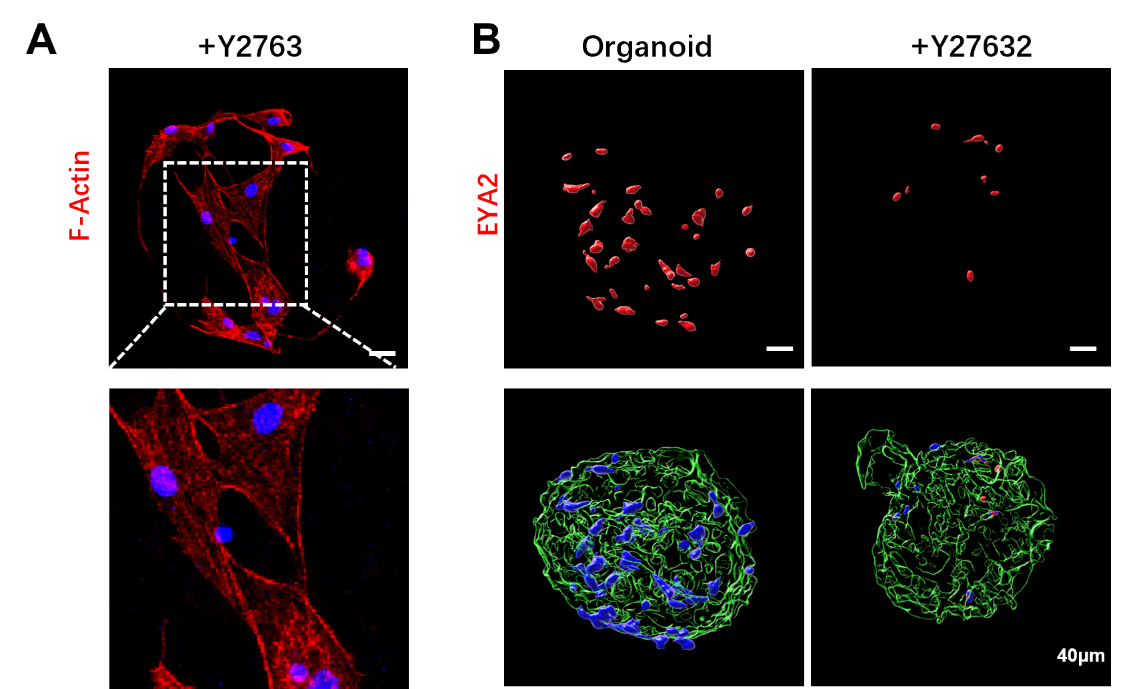


Fig. S6. organoid activates the RHO signaling pathway to promote tissue formation. (A) The organoid skeleton morphology after adding Y27632. (B) Confocal images of Eya2 immunofluorescent staining between DMEM and Y27632 treatment.


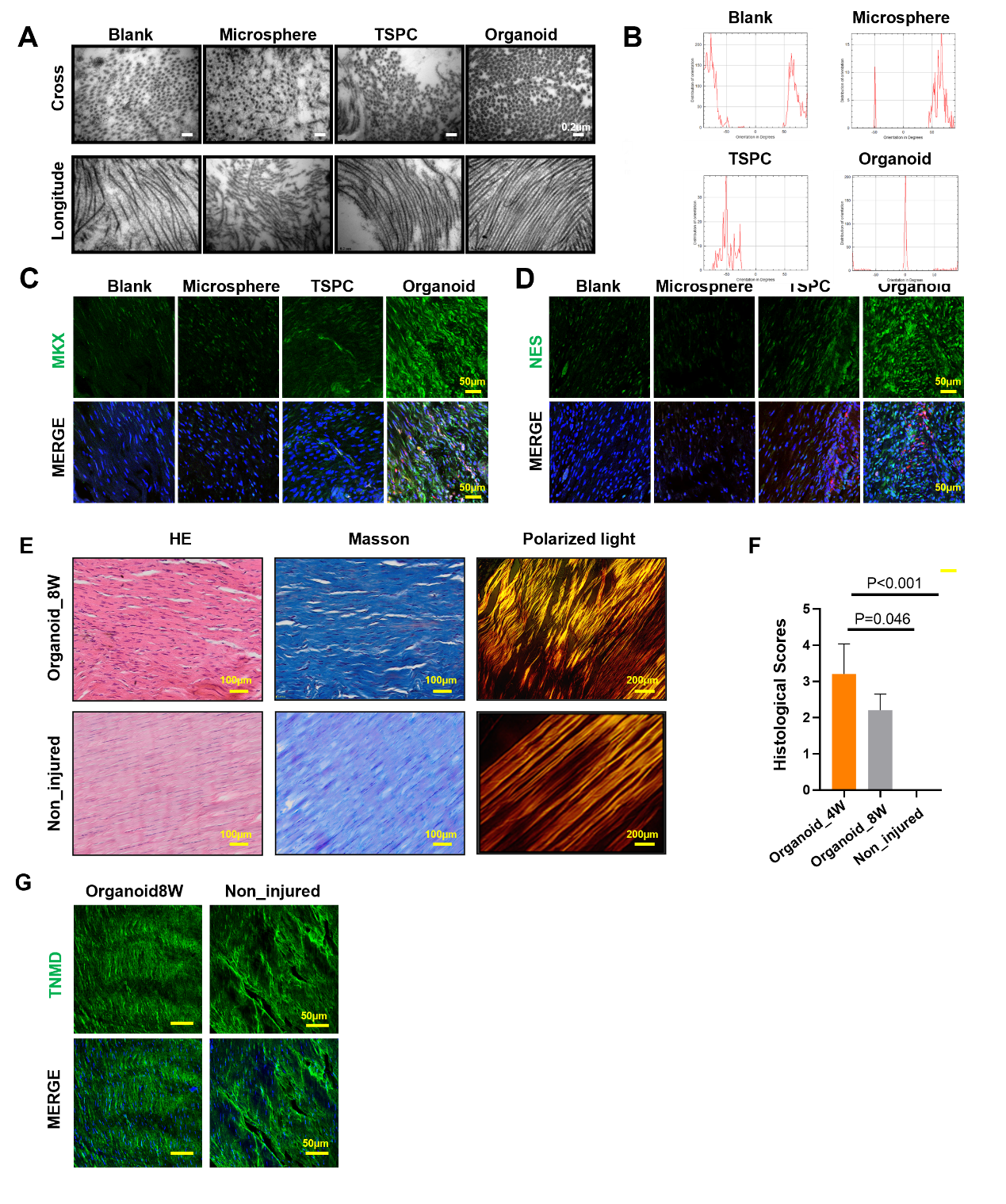


Fig. S7. hTSPCs organoid exhibited an enhanced tendon regeneration capacity at 2 weeks after surgery. (A, B) TEM of cross sections and longitudinal sections of the repaired collagen fibrils. Scale bars: 200 nm. (C, D) Immunofluorescence staining for MKX and NES in the repaired regenerated tendon at 4 weeks after surgery. Scale bars: 50 μm. (D, E) (F, G) Immunostaining for key tendon markers, such as TNMD and COL1.


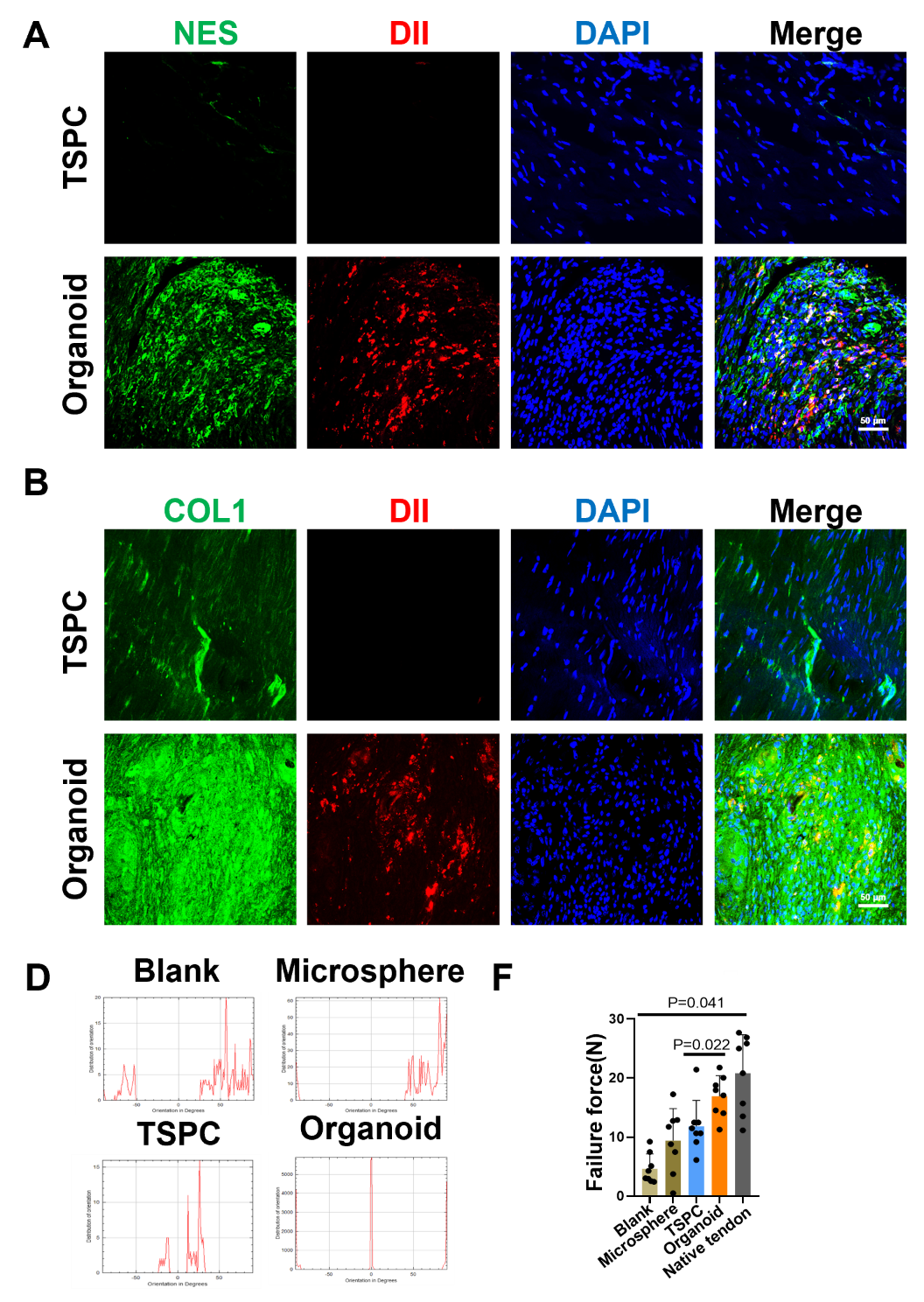


Fig. S8. hTSPCs organoid exhibited an enhanced tendon regeneration capacity at 4 weeks after surgery. (A-C) Immunofluorescence of NES, MKX and COL1 between organoid group and TSPC group in regeneration area at 4 weeks after surgery. (D) The angle distribution in TEM images of cross sections and longitudinal sections of the repaired collagen fibrils.


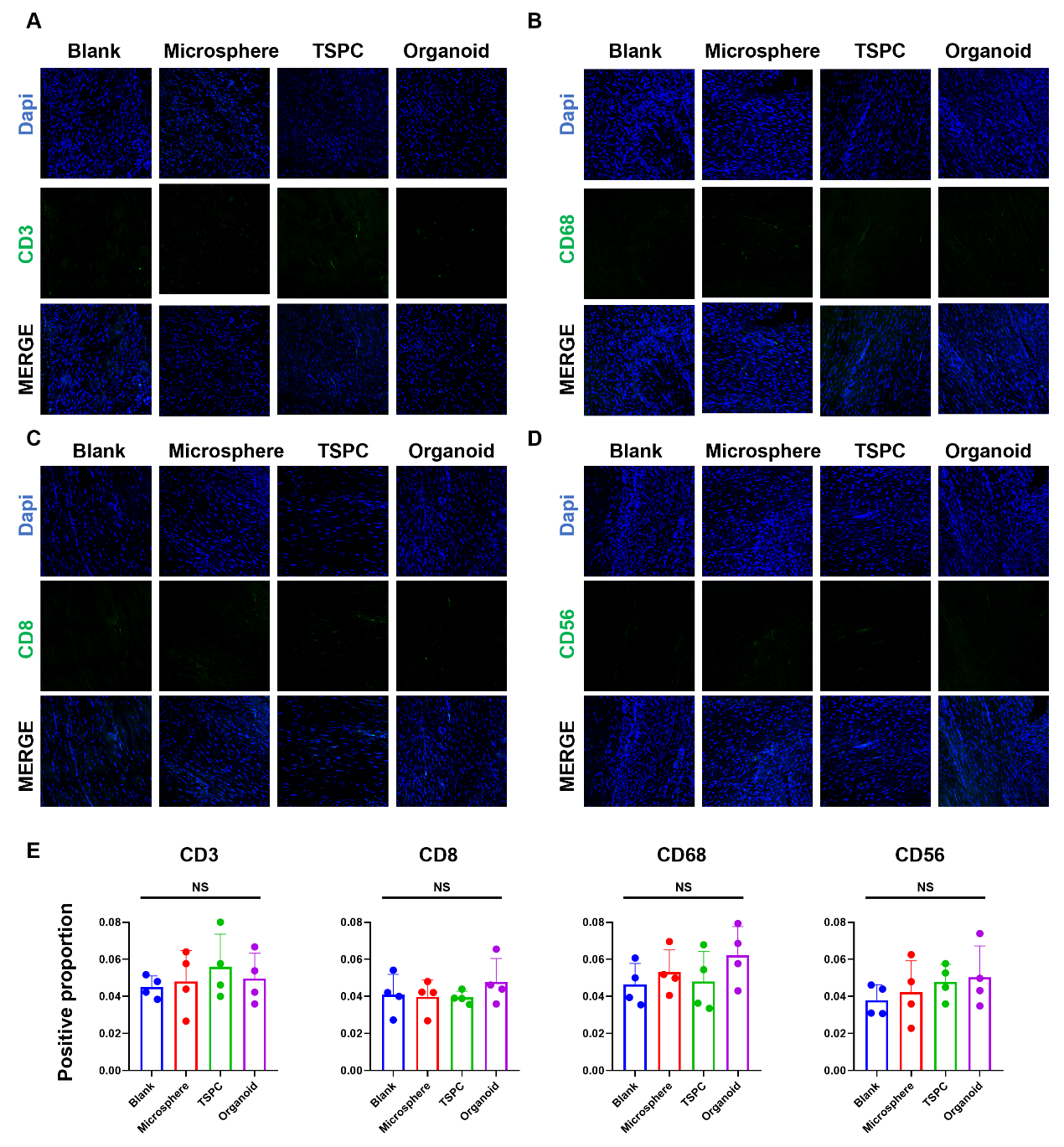


Fig. S9. Immunofluorescence analysis of CD3, CD8, CD56, and CD68 reveals low immunogenic response at 2 weeks.

Table S1. Materials.

| **REAGENT or RESOURCE** | **SOURCE** | **IDENTIFIER** |
| --- | --- | --- |
| **Antibodies** |  |  |
| Anti-Collagen I Antibody | Abcam | ab34710 |
| Anti-tenomodulin Antibody | Abcam | ab81328 |
| Anti-MKX Antibody | lifespan | LS-B8063-50 |
| Anti-Laminb Antibody | Abcam | ab16048 |
| Mki-67 | Abcam | ab16667 |
| MTOR | CST | 5536s |
| EGR1 | Proteintech | 22008-1-AP |
| EYA2 | Sigma | HPA027024 |
| Anti-COL14A1 Antibody | Abcam | ab58084 |
| Anti-Nestin Antibody, clone rat-401 | MERCK | MAB353 |
| c-Fos (9F6) Rabbit mAb | Cell Signaling Technology | 2250 |
| Anti-SCXA antibody | Abcam | ab58655 |
| Scleraxis (A-7) | Santa Cruz | sc-518082 |
| CD105 (Endoglin) Monoclonal Antibody (SN6), PE | eBioscience™ | 12-1057-42 |
| APC anti-human CD90 (Thy1) Antibody | Biolegend | 328113 |
| APC anti-human CD44 Antibody | Biolegend | 338805 |
| APC anti-human CD34 Antibody | Biolegend | 343607 |
| PE anti-human CD18 Antibody | Biolegend | 373407 |
| PE anti-human CD54 Antibody | Biolegend | 322707 |
| DAPI | beyotime | C1002 |
| **Chemicals, Peptides, and Recombinant Proteins** | | |
| Human KGF | peprotech | 100-19-10 |
| AZD4547 | Selleck | S2801 |
| Ascorbic acid 2-phosphate | Sigma | A8960 |
| DMEM, Low Glucose, Pyruvate | Gibco | 11885092 |
| DMEM, High Glucose | Gibco | 11965118 |
| FBS | Gibco | 10099-141 |
| PS | Gibco | 15140122 |
| Trypsin-EDTA | Gibco | 15400054 |
| Calcein-AM/PI Double Stain Kit | Yeasen | 40747ES76 |
| CellTracker TM CM-DiI Dye | Invitrogen | C7000 |
| Actin Cytoskeleton / Focal Adhesion Staining Kit | Merck | FAK100 |
| BCIP/NBT Alkaline Phosphatase Color Development Kit) | beyotime | C3206 |
| Direct red 80 | BBI Life Sciences | A606358-0025 |
| Alizarin Red S | sigma | A5533 |
| MesenCult™ Adipogenic Differentiation Kit (Human) | StemCell Technologies | 05412 |
| Oil Red O solution | sigma | O1391 |
| **Experimental Models** |  |  |
| hTSPCs | Primary cell isolation | \ |
| SD Rat | Slac | SPF |
| **Other materials** |  |  |
| 3D FloTrixTM expansion kit (3D gelatin microcarriers and 3D microcarriers digest solution) | Cytoniche | FK01-100 |
| **Software and Algorithms** |  |  |
| R | The R Project | https://www.r-project.org/ |
| ImageJ | \ | https://imagej.net/Welcome |

Table S2. qRT-PCR primers.

| Genes | 5’-3’ | Primer |
| --- | --- | --- |
| Human  CD146 | Forward | AGAGCCAACAGCACCTCCACA |
|  | Reverse | CTGGGAGCTTATCTGACTTAACTTC |
| Human  CD105 | Forward | CAACATGCAGATCTGGACCAC |
|  | Reverse | CTTTAGTACCAGGGTCATGGC |
| Human  CD90 | Forward | ATGAAGGTCCTCTACTTATCCGC |
|  | Reverse | GCACTGTGACGTTCTGGGA |
| Human  CD44 | Forward | TGCCTTTGATGGACCAATTACC |
|  | Reverse | GGACTGTCTTCGTCTGGGATGG |
| Human  CDK1 | Forward | GATTCTATCCCTCCTGGTC |
|  | Reverse | AATATGGTGCCTATACTCC |
| Human  KI67 | Forward | GCCTGCTCGACCCTACAGA |
|  | Reverse | GCTTGTCAACTGCGGTTGC |
| Human  PCNA | Forward | GGGCGTGAACCTCACCAGTA |
|  | Reverse | CAAGGTATCCGCGTTATCTTCG |
| Human  SCX | Forward | CGAGAACACCCAGCCCAAAC |
|  | Reverse | CTCCGAATCGCAGTCTTTCTGTC |
| Human  FOS | Forward | GAGGAGGGAGCTGACTGATAC |
|  | Reverse | CCAGGTCATCAGGGATCTTGCA |
| Human  DCN | Forward | TCAATGGACTGAACCAGATGA |
|  | Reverse | CCTTGAGGAATGCTGGTGAT |
| Human  TNMD | Forward | TGGGTGGTCCCTCAAGTGAAAGT |
|  | Reverse | CTCGACGGCAGTAAATACAACAATA |
| Human  FMOD | Forward | CAATGTCTACACCGTCCCTGA |
|  | Reverse | AGAAGGCTGCTGGAGTTGAAG |
| Human  EGR1 | Forward | CACATCCCTGCTAAAGCTGAA |
|  | Reverse | GTCGAAACGGCCATAGATGAT |
| Human  COL3A1 | Forward | TTTTGCAGTGATATGTGATGTT |
|  | Reverse | GGATGGTGGTTTTCAGTTTA |
| Human  GAPDH | Forward | TGACGCTGGGGCTGGCATTG |
|  | Reverse | GGCTGGTGGTCCAGGGGTCT |
